# Five new mexicanolide type limonoids from *Heynea trijuga*

**DOI:** 10.1007/s13659-012-0040-1

**Published:** 2012-06-11

**Authors:** Wei Yang, Ling-Mei Kong, Shi-Fei Li, Yan Li, Yu Zhang, Hong-Ping He, Xiao-Jiang Hao

**Affiliations:** 1 State Key Laboratory of Phytochemistry and Plant Resources in West China, Kunming Institute of Botany, Chinese Academy of Sciences, Kunming, 650201 China; 2 Graduate University of Chinese Academy of Sciences, Beijing, 100049 China

**Keywords:** *Heynea trijuga*, Meliaceae, limonoids, mexicanolide-type, heytrijunolide

## Abstract

Five new mexicanolide-type limonoids, heytrijunolides A–E (**1–5**) were isolated from the branches and leaves of *Heynea trijuga*. The structures of these new compounds were elucidated on the basis of extensive spectroscopic analysis. Compound **3** showed weak cytotoxicity against HL-60, SMMC-7721 and A-549 human tumor cell lines with the IC_50_ values of 21.88, 20.66 and 12.70 µM, respectively.

**Electronic Supplementary Material:**

Supplementary material is available for this article at 10.1007/s13659-012-0040-1 and is accessible for authorized users.

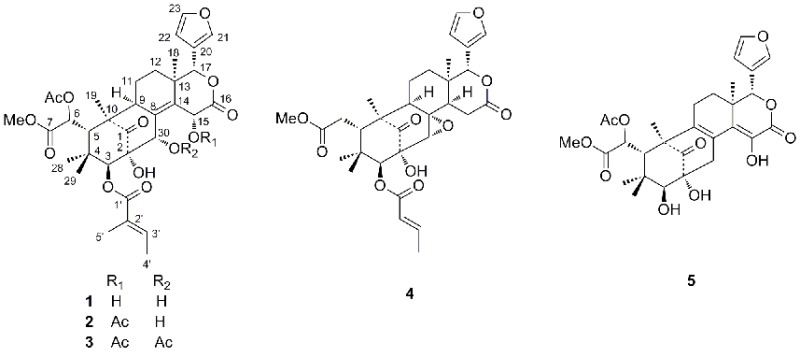

## Introduction

Limonoids are highly oxygenated and modified nortriterpenoids mainly found in the plants of the Meliaceae and Rutaceae families, which either containing or derived from a precursor with a 4,4,8-trimethyl-17-furanyl steroid skeleton, and have attracted continuous attention due to their diverse structures and significant biological activities.[1,2] The bioactivity, such as antimalarial, antimicrobial, cytotoxic, insects growth-regulating, insects antifeeding, insecticidal, and antiphytopathogen activities has been reported.[2] Till now, about 35 carbon frameworks have been isolated from Meliaceae family.[2] *Heynea trijuga* Roxburgh (previously named: *Trichilia connaroides* var. *microcarpa* Bentvelzen) (Meliaceae) is distributed mainly in southern of China.[3] Previous investigation on the chemical constituents of the genus *Heynea* has yielded a series of new limonoids, including trijugin-type, 30-nortrijugin-type, phragmalin-type, and mexicanolide-type.[4–12] In our continuing effort to search for novel limonoids from Meliaceae family, five new mexicanolide-type limonoids (**1**–**5**) were isolated from the branches and leaves of *H. trijuga* collected from Hainan province of China. Herein we describe the isolation, structural elucidation and bioactivity assays of these compounds.

## Results and Discussion

Heytrijunolide A (**1**) was isolated as white and amorphous powder. The molecular formula, $C_{34}H_{42}O_{13}$, was deduced from the positive HRESIMS ion at *m*/*z* = 681.2524 ($[M + Na]^+$, calcd for $C_{34}H_{42}O_{13}Na$, 681.2523). Its IR absorption bands showed the presence of hydroxyl (3442 $cm^{-1}$) and ketone groups (1728 $cm^{-1}$). The observation of proton signals for a $\beta$-substituted furan ring ($\delta_H$ 7.58 (1H, s, H-21), 6.48 (1H, s, H-22), and 7.44 (1H, s, H-23)), a methoxy group ($\delta_H$ 3.75, 3H, s), four tertiary methyls ($\delta_H$ 1.01 (3H, s, H-18), 1.25 (3H, s, H-19), 1.06 (3H, s, H-28), and 0.83 (3H, s, H-29)), and a characteristic low-field H-17 proton at $\delta$ 5.38 (1H, s) in the $^1H$ NMR spectrum, as well as the characteristic carbonyl group at C-1 ($\delta_C$ 213.0) in the $^{13}C$ NMR spectrum, strongly suggested that **1** was a mexicanolide-type limonoid.[13,14] The $^1H$ and $^{13}C$ NMR data (Tables 1 and 2) of **1** including the fully substituted olefinic resonances at $\delta_C$ 135.8 and 140.4 due to C-8 and C-14, respectively, were similar to those of augustineolide.[15] The major differences between them were the absence of isobutyryl group in compound **1**, and the locations of the substituent. Detailed analysis of the 2D NMR spectra (HSQC, $^1H$-$^1H$ COSY, and HMBC) of compound **1**, especially the key HMBC cross-peaks of H-3 ($\delta_H$ 5.02, 1H, s)/C-1′ ($\delta_C$ 166.8), H-6 ($\delta_H$ 5.46 (1H, s))/C-1″ ($\delta_C$ 170.0), OH-2 ($\delta_H$ 4.23 (1H, s))/C-1, C-2 ($\delta_C$ 79.4), C-3 ($\delta_C$ 85.8), OH-15 ($\delta_H$ 3.42 (1H, s))/C-14 ($\delta_C$ 140.4), C-15 ($\delta_C$ 65.2), C-16 ($\delta_C$ 173.9), and OH-30 (2.58

*To whom correspondence should be addressed. E-mail: hehongping@mail.kib.ac.cn (H.P. He); zhangyu@mail.kib.ac.cn (Y. Zhang).

**Table 1. [1]H NMR spectral data of compounds 1–5 in $CDCl_3$ (*J* in Hz)**

| pos. | 1[a] | 2[a] | 3[b] | 4[a] | 5[a] |
|---|---|---|---|---|---|
| 3 | 5.02, s | 4.89, s | 5.02, s | 5.11, s | 4.03, s |
| 5 | 3.58, s | 3.55, s | 3.59, s | 3.14, dd (9.4, 1.9) | 2.95, s |
| 6 | 5.46, s | 5.42, s | 5.48, s | 2.31, m | 5.46, s |
| 9 | 2.45, d (8.2) | 2.37, d (7.3) | 2.41, s | 1.87, m | |
| 11*α* | 1.80, m | 1.84, m | 1.12, d (6.3) | 1.77, m | 2.20, m |
| 11*β* | 1.98, br. d (14.8) | 1.92, m | 1.82, m | 1.87, m | 2.29, d (18. 9) |
| 12*α* | 1.11, br. d (13.0) | 1.12, d (13.5) | 1.17, m | 1.18, m | 1.52, dd (12.6, 3.6) |
| 12*β* | 1.87, overlap | 1.82, m | 1.87, m | 1.96, m | 1.36, m |
| 14 | | | | 1.60, m | |
| 15*α* | | | | 2.83, dd (16.1, 4.9) | |
| 15*β* | 5.02, s | 6.37, s | 6.52, s | 3.55, dd (16.1, 1.8) | |
| 17 | 5.38, s | 5.44, s | 5.59, s | 5.17, s | 5.15, s |
| 18 | 1.01, s | 1.06, s | 1.07, s | 0.98 , s | 1.00, s |
| 19 | 1.25, s | 1.21, s | 1.29, s | 1.14, s | 1.24, s |
| 21 | 7.58, s | 7.55, s | 7.59, s | 7.47, s | 7.49, s |
| 22 | 6.48, s | 6.44, s | 6.47, s | 6.43, br. s | 6.44, s |
| 23 | 7.44 , s | 7.38, s | 7.42, s | 7.41, br. s | 7.45, s |
| 28 | 1.06, s | 0.98, s | 1.02, s | 0.75, s | 1.26, s |
| 29 | 0.83, s | 0.82, s | 0.85, s | 0.77, s | 1.16, s |
| 30*α* | | | | | 2.85, dd (18.5, 3.1) |
| 30*β* | 4.79, s | 4.25, s | 5.66, s | 3.48, s | 4.22, d (18.5) |
| 7-OMe | 3.75, s | 3.72, s | 3.76, s | 3.71, s | 3.75, s |
| 2-OH | 4.23, s | | | | 3.99, s |
| 3-OH | | | | | 2.42, s |
| 15-OH | 3.42, s | | | | |
| 30-OH | 2.58, br. s | | | | |
| 2′ | | | | 6.02, dq (15.6, 1.7) | |
| 3' | 6.86, q (7.0) | 6.78, q (6.9) | 6.77, q (6.6) | 7.16, dq (15.6, 6.9) | |
| 4' | 1.77, d (7.0) | 1.74, d (6.9) | 1.77, d (6.6) | 1.98, dd (6.9, 1.7) | |
| 5' | 1.87, s | 1.91, s | 1.98, s | | |
| 6-Ac | 2.17, s | 2.12, s | 2.17, s | | 2.06, s |
| 15-Ac | | 2.07, s | 2.02, s | | |
| 30-Ac | | | 1.96, s | | |

[a]600 MHz; [b]400 MHz

(1H, br. s))/C-2, C-8 ($\delta_C$ 135.8), C-30 ($\delta_C$ 73.9) indicated that the tigloyl, acetoxy, and three hydroxy groups were placed at C-3, C-6, C-2, C-15, and C-30, respectively.

The relative configuration of **1** was deduced from the analysis of its ROESY correlations. As shown in Figure 1, the observed ROESY correlations of Me-29/H-5, H-5/H-12*β*, H-12*β*/H-17, H-17/H-15, H-17/H-3′, and H-15/H-30 indicated that these protons and the C-3 tigloyl group were all *β*-oriented, whereas the ROESY correlations of Me-28/H-3, Me-28/Me-19, Me-19/H-9, H-9/H-11*α*, and H-11*α*/Me-18 revealed their *α*-orientations. Therefore, the structure of compound **1** was finally established.

**Figure 1.** Selected 2D NMR correlations of **1**

Compound **2** was determined to be the 15-*O*-acetyl derivative of **1** according to the following information. Comparing the NMR (Tables 1 and 2) and MS data of **1** and **2**, one more acetyl group was present in **2**. The acetoxy group was located at C-15 in **2** on the basis of the HMBC correlation of H-15 ($\delta_H$ 6.37 (1H, s))/Ac-15 ($\delta_C$ 169.3). Furthermore, compound **3** possessed the molecular formula $C_{38}H_{46}O_{15}$ as determined by positive HRESIMS, with 42 mass units more than that of **2**. Detailed studies of its 1D and 2D NMR spectra indicated that **3** was the 30-*O*-acetyl derivative of **2**. Moreover, this was confirmed by the HMBC correlation of H-30 ($\delta_H$ 5.66 (1H, s))/Ac-30 ($\delta_C$ 168.1).

Heytrijunolide D (**4**) was obtained as a white amorphous powder. The positive HRESIMS displayed a molecular formula, $C_{31}H_{38}O_{10}$ by the ion peak at *m/z* 593.2362 ([M + Na]$^+$, calcd for $C_{31}H_{38}O_{10}Na$, 593.2362). Inspection of the [1]H and [13]C NMR spectra revealed the characteristic NMR resonances of mexicanolide type limonoids [13,14] with a furan ring ($\delta_H$ 7.47 (1H, s, H-21), $\delta_C$ 141.2, C-21; 7.41 (1H, br. s, H-23), $\delta_C$ 143.4, C-23; 6.43 (1H, br. s, H-22), $\delta_C$ 110.4, C-22; and $\delta_C$ 120.3, C-20), C-7 carbomethoxy ester ($\delta_H$ 3.71 (3H, s), $\delta_C$ 52.7; $\delta_C$ 174.3, C-7), four quaternary methyl singlet resonances, as well as the carbonyl group at C-1 ($\delta_C$ 213.5). Comparison of its spectroscopic data with those of 3-angeloyl-3-detigloylruageanin B[16] showed a close similarity, suggesting that **4** was an analogue of the latter. And the main difference between them was that an (*E*)-*α*,*β*-unsaturated butyroxy located at C-3 replaced an angeloyl in compound **4**, which was further confirmed by the HMBC cross-peak of H-3 ($\delta_H$ 5.11 (1H, s))/C-1′ ($\delta_C$ 165.4). Moreover, the coupling constants of H-2′ (dd, *J* = 15.6, 1.7 Hz) and H-3' (dq, *J* = 15.6, 6.9 Hz) revealed an *E*-geometry for the $\Delta^{2'(3')}$ double bond. The ROESY experiments indicated that the relative configuration of **4** was the same as that of 3-angeloyl-3-detigloylruageanin B.

**Table 2.** $^{13}C$ **NMR spectral data of compounds 1–5 in $CDCl_3$**

| pos. | **1**[a] | **2**[a] | **3**[b] | **4**[a] | **5**[a] |
|---|---|---|---|---|---|
| 1 | 213.0, C | 212.8, C | 211.8 , C | 213.5, C | 213.0, C |
| 2 | 79.4, C | 79.5, C | 78.8, C | 78.3, C | 78.2, C |
| 3 | 85.8, CH | 86.4, CH | 86.0, CH | 84.7, CH | 83.5, CH |
| 4 | 40.4, C | 40.2, C | 40.1, C | 40.1, C | 39.5, C |
| 5 | 44.6, CH | 44.9, CH | 44.6, CH | 42.5, CH | 55.4, CH |
| 6 | 72.9, CH | 72.8, CH | 72.4, CH | 33.0, $CH_2$ | 70.2, CH |
| 7 | 171.8, C | 171.7, C | 171.4, C | 174.3, C | 170.9, C |
| 8 | 135.8, C | 134.8, C | 132.5, C | 63.2, C | 127.2, C |
| 9 | 46.3, CH | 47.0, CH | 48.1, CH | 55.5, CH | 142.8, C |
| 10 | 52.7, C | 52.4, C | 52.2, C | 49.2, C | 52.1, C |
| 11 | 18.1, $CH_2$ | 18.2, $CH_2$ | 18.0, $CH_2$ | 19.5, $CH_2$ | 22.3, $CH_2$ |
| 12 | 28.2, $CH_2$ | 28.5, $CH_2$ | 28.4, $CH_2$ | 33. 5, $CH_2$ | 29.9, $CH_2$ |
| 13 | 39.3, C | 39.1, C | 39.1, C | 36.5, C | 37.7, C |
| 14 | 140.4, C | 140.3, C | 139.7, C | 45.6, C | 124.3, C |
| 15 | 65.2, CH | 63.9, CH | 64.3, CH | 33.8, $CH_2$ | 134.7, C |
| 16 | 173.9, C | 167.7, C | 167.5, C | 172.0, C | 165.9, C |
| 17 | 81.5, CH | 81.0, CH | 80.0, CH | 79.0, CH | 81.1, CH |
| 18 | 16.2, $CH_3$ | 16.7, $CH_3$ | 17.0, $CH_3$ | 26.5, $CH_3$ | 17.0, $CH_3$ |
| 19 | 17.2, $CH_3$ | 17.2, $CH_3$ | 16.9, $CH_3$ | 16.4, $CH_3$ | 18.6, $CH_3$ |
| 20 | 120.2, C | 120.5, C | 120.1, C | 120.3, C | 119.5, C |
| 21 | 141.9, CH | 142.1, CH | 141.8, CH | 141.2, CH | 141.4, CH |
| 22 | 110.0, CH | 110.1, CH | 109.7, CH | 110.4, CH | 110.0, CH |
| 23 | 143.5, CH | 143.4, CH | 143.0, CH | 143.4, CH | 143.3, CH |
| 28 | 22.4, $CH_3$ | 22.6, $CH_3$ | 22.1, $CH_3$ | 22.1, $CH_3$ | 25.3, $CH_3$ |
| 29 | 23.2, $CH_3$ | 23.4, $CH_3$ | 23.0, $CH_3$ | 20.4, $CH_3$ | 28.4, $CH_3$ |
| 30 | 73.9, CH | 73.4, CH | 73.9, CH | 67.8, CH | 41.9, $CH_2$ |
| 7-OMe | 53.6, $CH_3$ | 53.7, $CH_3$ | 53.3, $CH_3$ | 52. 7, $CH_3$ | 52.9, $CH_3$ |
| 1' | 166.8, C | 166.8, C | 166.6, C | 165.4, C | |
| 2' | 129.6, C | 130.7, C | 130.9, C | 121.3, CH | |
| 3' | 138.5, CH | 137.9, CH | 136.9, CH | 148.5, CH | |
| 4' | 14.6, $CH_3$ | 14.7, $CH_3$ | 14.2, $CH_3$ | 18.6, $CH_3$ | |
| 5' | 12.8, $CH_3$ | 12.7, $CH_3$ | 12.6, $CH_3$ | | |
| Ac-6 (1") | 170.0, C | 169.9, C | 169.6, C | | 169.2, C |
| 2" | 21.2, $CH_3$ | 21.2, $CH_3$ | 20.8, $CH_3$ | | 20.6, $CH_3$ |
| Ac-15 | | 169.3, C | 169.4, C | | |
| | | 20.8, $CH_3$ | 21.1, $CH_3$ | | |
| Ac-30 | | | 168.1, C | | |
| | | | 20.7, $CH_3$ | | |

[a]150 MHz; [b]100 MHz

Heytrijunolide E (**5**) was isolated as a white, amorphous powder. The molecular formula was assigned as $C_{29}H_{34}O_{11}$ from its HRESIMS peak at *m/z* 581.1994 ($[M + Na]^+$, calcd for $C_{29}H_{34}O_{11}Na$, 581.1998). The IR spectrum showed strong absorption bands at 3432, 1762, and 1713 $cm^{-1}$, suggesting the presence of hydroxyl, and carbonyl. $^1H$, $^{13}C$, and DEPT NMR data of **5** (Tables 1 and 2) revealed a $\beta$-furan moiety, four methyl singlets, a carbomethoxy group, and a keto carbonyl at C-1 ($\delta_C$ 213.0). The information above strongly suggested that **5** was a mexicanolide-type limonoid.[13,14] The key HMBC cross-peaks of H$\beta$-30/C-8, C-9, H-18/C-13, C-14 and H-17/C-16 revealed a conjugated vinyl-vinyl-lactone unit from C-9 to C-16. The planar structure of **5** was further confirmed by detailed 2D NMR analysis (Figure 2). An acetoxy group was located at C-6 by the HMBC correlation of H-6/C-1″, and two hydroxyls were located at C-2 and C-3 by the HMBC correlations of OH-3 ($\delta_H$ 2.42 (1H, s))/C-3 ($\delta_C$ 83.5), C-4 ($\delta_C$ 39.5), and OH-2 ($\delta_H$ 3.99 (1H, s))/C-1 ($\delta_C$ 213.0), C-2 ($\delta_C$ 78.2), C-3 ($\delta_C$ 83.5). Furthermore, there was one more hydroxyl in **5** according to its molecular formula. The $^{13}C$ and DEPT spectra totally revealed five olefinic quaternary carbons, but only four of those were already established in the structural segment. So the remaining hydroxy group must be located at C-15. Therefore, the hydroxyl and the conjugated vinyl-vinyl-lactone unit formed a conjugated vinyl-enol-lactone unit.

The relative configuration of **5** was deduced from the analysis of its ROESY correlations. As shown in Figure 2, the observed correlations of Me-29/H-5, H-5/H-11$\beta$, H-11$\beta$/H-12$\beta$, and H-12$\beta$/H-17 indicated that these protons were all $\beta$-oriented. Furthermore, the observed correlations of Me-28/H-3, H-12$\alpha$/Me-18, and H-11$\alpha$/Me-19 indicated that these protons were all $\alpha$-oriented. Therefore, the structure of **5** was established.

**Figure 2.** Selected 2D NMR correlations of **5**

Compounds **1** and **3** were selected to evaluate insecticidal activity for *Artemia salina* L. (brine shrimp).[17] The results showed that **3** displayed activity at 100 ppm, with the corrected mortality 64.96%. Moreover, compound **3** was further tested *in vitro* for inhibitory activities against the HL-60, SMMC-7721, A-549, MCF-7, and SW480 human tumor cell lines, using the MTT method.[18] The results indicated **3** had weak cytotoxicity against HL-60, SMMC-7721 and A-549 cells with the $IC_{50}$ values of 21.88, 20.66 and 12.70 $\mu$M, respectively.

## Experimental Section

**General Experimental Procedures.** Optical rotations were measured with a JASCO P-1020 digitsl polarimeter. UV spectrawere recorded with a Shimadzu UV-2401 PC spectrophotometer. IR spectra were recorded on a Bruker Tensor-27 infrared spectrometer with a KBr disk. $^1$H and $^{13}$C NMR spectra were recorded on a Bruker AM-400 spectrometer and 2D NMR spectra were recorded on a Bruker DRX-500 instrument and a Bruker Avance-600 spectrometer. Chemical shifts were reported using TMS as the internal standard. ESIMS, HRESIMS and HREIMS spectra were measured with a Brucker HCT Esquire 3000, API QSTAR Pulsarspectrometer and Waters Auto Premier P776 spectrom, respectively. Column chromatography was performed on silica gel (90–150 $\mu$m; Qingdao Marine Chemical Inc.), Sephadex LH-20 (40–70 $\mu$m; Amersham Pharmacia Biotech AB, Uppsala, Sweden), and Lichroprep RP-18 gel (20–45 $\mu$m; Merck, Darmstadt, Germany). Precoated silica gel $GF_{254}$ and $HF_{254}$ plates (Qindao Haiyang Chemical Plant, Qingdao, China) were used for thin-layer chromatography. Semipreparative HPLC was performed on a Hypersil gold column (i.d. 10 × 250 mm; thermo fisher scientific Co., Ltd). Fractions were monitored by TLC, and spots were visualized by heating thin-layer chromatography sprayed with 10% $H_2SO_4$.

**Plant Materal.** The branches and leaves of *H. trijuga* Roxburgh were collected from Changjiang County, Hainan Province, China in December, 2010. The plant was identified by Dr. Guangwan Hu (Kunming Institute of Botany, Chinese Academy of Sciences). And its voucher specimen (H20101203) was deposited at the State Key Laboratory of Phytochemistry and Plant Resources in West China, Kunming Institute of Botany, CAS.

**Extraction and Isolation.** The air-dried powder of the plant material (12.0 kg) was extracted three times with 90% EtOH (25 L × 3, 4 h/time) under reflux to give a crude extract, which was suspended in water and then extracted successively with petroleum ether (PE) (8 L × 3), EtOAc (8 L × 6) to give two parts. The EtOAc part (180.0 g) was separated on a silica gel column (100–200 mesh, 10 × 100 cm, 1.0 kg) eluted with petroleum ether-$Me_2CO$ (100:0→0:100, each 20 L) to give seven fractions (Fr. 1→Fr. 7). After decoloration of Fr. 4 (35.3g) by MCI chromatography (75–150 $\mu$m) eluted with gradient MeOH-$H_2O$ (20% to 100%, each 10L), all fractions were detected by TLC. The fraction eluted with 80% (Fr. 4E) MeOH-$H_2O$ was detected containing Limonoids. Fr. 4E (7.0g) was purified by Sephadex LH-20 (eluted by $CHCl_3$-MeOH 1:1, 3.2 × 140 cm) to get three fractions (Fr. 4E1→Fr. 4E3). The fraction Fr. 4E1 (2.3g) purified by Sephadex LH-20 (eluted by MeOH, 2.0 × 140 cm) and further by semipreparative HPLC to afford **3** (120.3 mg, MeOH-$H_2O$, 63:37). The fraction Fr. 4E2 (1.0g) purified by Sephadex LH-20 (eluted by MeOH, 2.0 × 140 cm), RP-18 Si gel column (20-45 $\mu$m, 2 × 40 cm, 20 g) using a gradient system of Acetone-$H_2O$ (V/V = 10:90, 30:70, 50:50, 70:30, 90:10 each 4L) and semipreparative HPLC eluted with MeOH-$H_2O$ to produce compounds **1** (6.4 mg, MeOH-$H_2O$, 65:35), **2** (2.2 mg, MeOH-$H_2O$, 60:40), **4** (4.1 mg, MeOH-$H_2O$, 70:30), and **5** (1.2 mg, MeOH-$H_2O$, 60:40).

**Heytrijunolide A (1):** white powder; $[\alpha]_D^{15}$ – 62.4 (*c* 0.11, $CHCl_3$); UV (MeOH) $\lambda_{max}$ (log $\varepsilon$): 290 (2.19), 210 (3.59) nm; IR (KBr) $\nu_{max}$ 3442, 2956, 2923, 2853, 1728 cm$^{-1}$; $^1$H NMR (600 MHz, $CDCl_3$), see Table 1, $^{13}$C NMR (150 MHz, $CDCl_3$), see Table 2; positive-ion ESIMS, *m/z* 681 [M + Na]$^+$; HRESIMS, *m/z* 681.2524 ([M + Na]$^+$, calcd for $C_{34}H_{42}O_{13}Na$, 681.2523).

**Heytrijunolide B (2):** white powder; $[\alpha]_D^{22}$ – 48.8 (*c* 0.22, $CH_3OH$); UV (MeOH) $\lambda_{max}$ (log $\varepsilon$): 211 (3.50) nm; IR (KBr) $\nu_{max}$ 3439, 2929, 1757, 1728 cm$^{-1}$; $^1$H NMR (600 MHz, $CDCl_3$), see Table 1, $^{13}$C NMR (150 MHz, $CDCl_3$), see Table 2; positive-ion ESIMS, *m/z* 723 [M + Na]$^+$; HREIMS, *m/z* 700.2729 ([M]$^+$, calcd for $C_{36}H_{44}O_{14}$, 700.2731).

**Heytrijunolide C (3):** white powder; $[\alpha]_D^{21}$ – 65.42 (*c* 0.24, $CHCl_3$); UV (MeOH) $\lambda_{max}$ (log $\varepsilon$): 211 (3.69) nm; IR (KBr) $\nu_{max}$ 3455, 2954, 1755 cm$^{-1}$; $^1$H NMR (400 MHz, $CDCl_3$), see Table 1, $^{13}$C NMR (100 MHz, $CDCl_3$), see Table 2; positive-ion ESIMS, *m/z* 765 [M + Na]$^+$; HRESIMS, *m/z* 765.2737 ([M + Na]$^+$, calcd for $C_{38}H_{46}O_{15}Na$, 765.2734).

**Heytrijunolide D (4):** white powder; $[\alpha]_D^{15}$ – 76.82 (*c* 0.17, $CHCl_3$); UV ($CH_3OH$) $\lambda_{max}$ (log $\varepsilon$): 208 (3.26) nm; IR (KBr) $\nu_{max}$ 3442, 2924, 1732 cm$^{-1}$; $^1$H NMR (600 MHz, $CDCl_3$), see Table 1, $^{13}$C NMR (150 MHz, $CDCl_3$), see Table 2; positive-ion ESIMS, *m/z* 593 [M + Na]$^+$; HRESIMS, *m/z* 593.2362 ([M + Na]$^+$, calcd for $C_{31}H_{38}O_{10}Na$, 593.2362).

**Heytrijunolide E (5):** white powder; $[\alpha]_D^{15}$ + 126.98 (*c* 0.10, $CHCl_3$); IR (KBr) $\nu_{max}$ 3432, 2924, 1762, 1751, 1713 cm$^{-1}$; $^1$H NMR (600 MHz, $CDCl_3$), see Table 1, $^{13}$C NMR (150 MHz, $CDCl_3$), see Table 2; positive-ion ESIMS, *m/z* 581 [M + Na]$^+$; HRESIMS, *m/z* 581.1994 ([M + Na]$^+$, calcd for $C_{29}H_{34}O_{11}Na$, 581.1998).

**Insecticidal Assay.**[17] The test compounds were dissolved in DMSO or water and then diluted with artificial seawater to the final concentrations of 100, 50, 10 ppm (mg/L), which were added to 96-well plates with each well of 15–25 *Artemia salina*. After cultivation at 28 °C for 24 h, the numbers of the dead *A. salina* were counted with a microscope. Each concentration was repeated in triplicate with toosendanin as the positive control. And the control group was treated in the same way without samples. The corrected mortality was calculated by the Abbot formula.

Corrected mortality = (the mortality of the *A. salina* with sample – the mortality of the *A. salina* of control group) / (1 – the mortality of the *A. salina* of control group) × 100%

**Cytotoxicity Assay.**[18] HL-60, SMMC-7721, A-549, MCF-7 and SW480 were cultured in RPMI 1640 or DMEM medium (Hyclone, USA) supplemented with 10% fetal bovine serum (Hyclone, USA) at 37 °C. The cytotoxicity assay was performed according to the MTT method. The $IC_{50}$ values were calculated by the Reed and Muench method. DDP was included as a positive control.

## Electronic Supplementary Material

Supplementary material is available in the online version of this article at http://dx.doi.org/ 10.1007/s13659-012-0040-1 and is accessible for authorized users.

## Acknowledgments

We thank Dr. Qiang Zhang, Northwest A & F University for insecticidal bioactivity testing. This work was financially supported by grants from the National Basic Research Program of China (973 Program, 2009CB522300 and 2009CB940900), National Natural Science Funding of China (31170332), and the Young Academic and Technical Leader Raising Foundation of Yunnan Province (2010CI047).

**Open Access** This article is distributed under the terms of the Creative Commons Attribution License which permits any use, distribution, and reproduction in any medium, provided the original author(s) and source are credited.

## Electronic supplementary material

Supplementary material, approximately 3.98 MB.
