# Supplementary material for: Five new mexicanolide type limonoids from Heynea trijuga
Source: Nat Prod Bioprospect. 2012 Jun 11;2(4):145–9. doi: 10.1007/s13659-012-0040-1 (PMC4131624; doi:10.1007/s13659-012-0040-1)
Supplement: Supplementary file 1 — Supplementary material, approximately 3.98 MB. [file 13659_2012_40_MOESM1_ESM.pdf]

Electronic Supplementary Material

Five new mexicanolide type limonoids from *Heynea trijuga*

Wei YANG,[a,b] Ling-Mei KONG,[a,b] Shi-Fei LI,[a,b] Yan LI,[a] Yu ZHANG,[a,*] Hong-Ping HE,[a,*] and Xiao-Jiang HAO[a]

[a]State Key Laboratory of Phytochemistry and Plant Resources in West China, Kunming Institute of Botany, Chinese Academy of Sciences, Kunming 650201, China

[b]Graduate University of Chinese Academy of Sciences, Beijing 100049, China

Received 24 May 2012; Accepted 4 June 2012

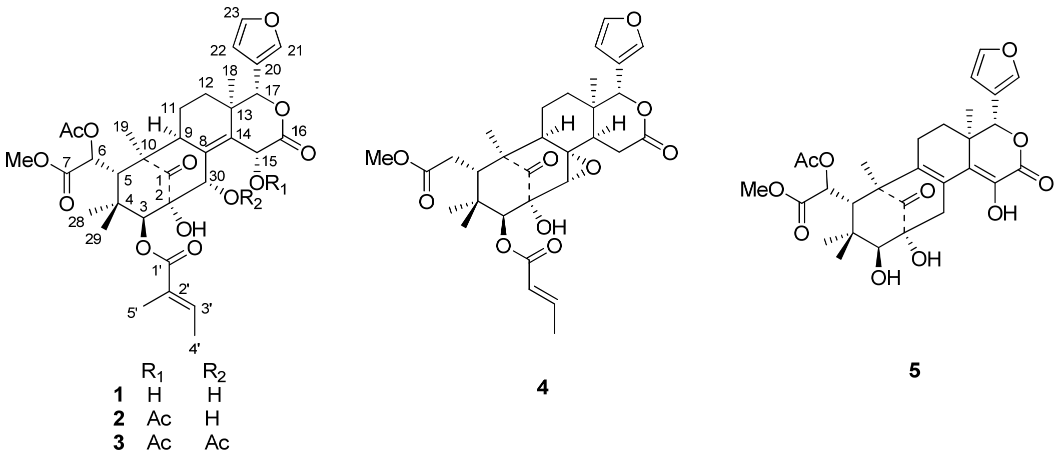

Structures of compounds **1**–**5**

*To whom correspondence should be addressed. E-mail: hehongping@mail.kib.ac.cn (H.P. He); zhangyu@mail.kib.ac.cn (Y. Zhang).

Legends for Figures

Fig. 1S ESIMS spectrum of compound **1**

Fig. 2S ^{1}H-NMR spectrum of compound **1** in $CDCl_3$

Fig. 3S ^{13}C-NMR spectrum of compound **1** in $CDCl_3$

Fig. 4S HSQC NMR spectrum of compound **1** in $CDCl_3$

Fig. 5S HMBC NMR spectrum of compound **1** in $CDCl_3$

Fig. 6S ^{1}H-^{1}H COSY NMR spectrum of compound **1** in $CDCl_3$

Fig. 7S ROESY NMR spectrum of compound **1** in $CDCl_3$

Fig. 8S IR spectrum of compound **1**

Fig. 9S UV spectrum of compound **1** in CH_3OH

Fig. 10S HREIMS spectrum of compound **2**

Fig. 11S ^{1}H-NMR spectrum of compound **2** in $CDCl_3$

Fig. 12S ^{13}C-NMR spectrum of compound **2** in $CDCl_3$

Fig. 13S HSQC NMR spectrum of compound **2** in $CDCl_3$

Fig. 14S HMBC NMR spectrum of compound **2** in $CDCl_3$

Fig. 15S ^{1}H-^{1}H COSY NMR spectrum of compound **2** in $CDCl_3$

Fig. 16S ROESY NMR spectrum of compound **2** in $CDCl_3$

Fig. 17S IR spectrum of compound **2**

Fig. 18S UV spectrum of compound **2** in CH_3OH

Fig. 19S HRESIMS spectrum of compound **3**

Fig. 20S ^{1}H-NMR spectrum of compound **3** in $CDCl_3$

Fig. 21S ^{13}C-NMR spectrum of compound **3** in $CDCl_3$

Fig. 22S HSQC NMR spectrum of compound **3** in $CDCl_3$

Fig. 23S HMBC NMR spectrum of compound **3** in $CDCl_3$

Fig. 24S ^{1}H-^{1}H COSY NMR spectrum of compound **3** in $CDCl_3$

Fig. 25S ROESY NMR spectrum of compound **3** in $CDCl_3$

Fig. 26S IR spectrum of compound **3**

Fig. 27S UV spectrum of compound **3** in CH_3OH

Fig. 28S ESIMS spectrum of compound **4**

Fig. 29S ^{1}H-NMR spectrum of compound **4** in $CDCl_3$

Fig. 30S ^{13}C-NMR spectrum of compound **4** in $CDCl_3$

Fig. 31S HSQC NMR spectrum of compound **4** in $CDCl_3$

Fig. 32S HMBC NMR spectrum of compound **4** in $CDCl_3$

Fig. 33S ^{1}H-^{1}H COSY NMR spectrum of compound **4** in $CDCl_3$

Fig. 34S ROESY NMR spectrum of compound **4** in $CDCl_3$

Fig. 35S IR spectrum of compound **4**

Fig. 36S UV spectrum of compound **4** in CH_3OH

Fig. 37S ESIMS spectrum of compound **5**

Fig. 38S ^{1}H-NMR spectrum of compound **5** in $CDCl_3$

Fig. 39S ^{13}C-NMR spectrum of compound **5** in $CDCl_3$

Fig. 40S HSQC NMR spectrum of compound **5** in $CDCl_3$

Fig. 41S HMBC NMR spectrum of compound **5** in $CDCl_3$

Fig. 42S ^{1}H-^{1}H COSY NMR spectrum of compound **5** in $CDCl_3$

Fig. 43S ROESY NMR spectrum of compound **5** in $CDCl_3$

Fig. 44S IR spectrum of compound **5**

Fig. 1S ESIMS spectrum of compound **1**

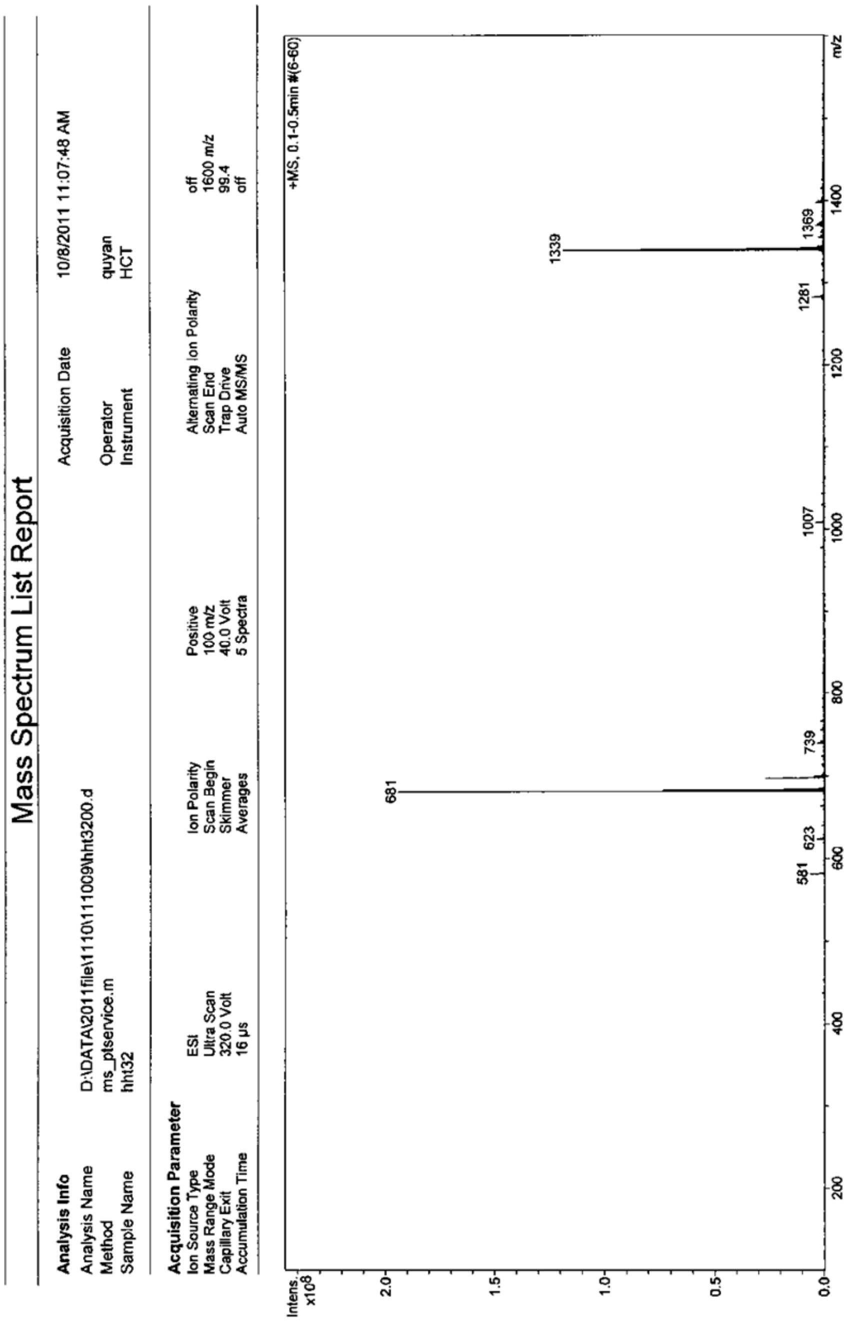

Fig. 2S ^{1}H-NMR spectrum of compound **1** in $CDCl_3$

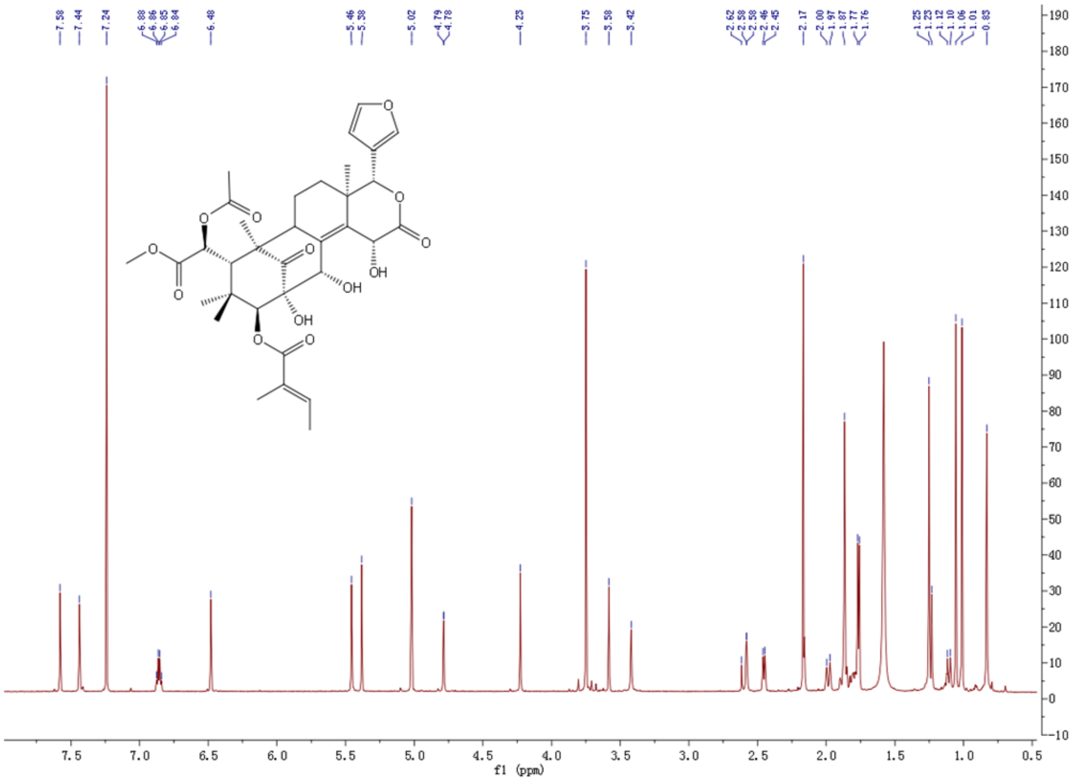

Fig. 3S ^{13}C-NMR spectrum of compound **1** in $CDCl_3$

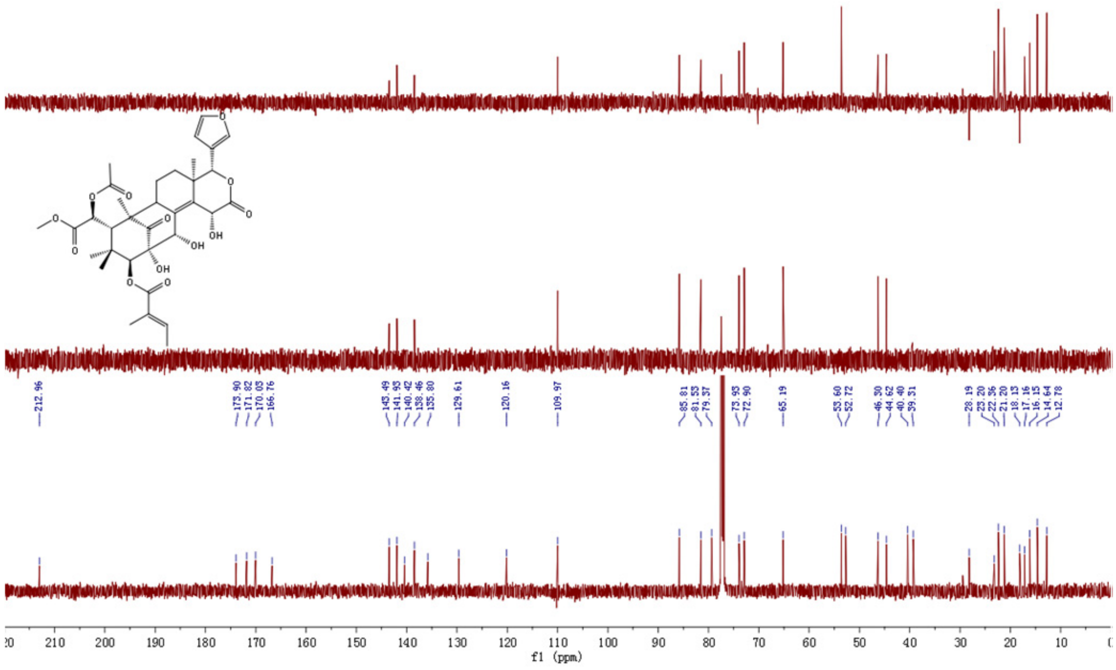

Fig. 4S HSQC NMR spectrum of compound **1** in $CDCl_3$

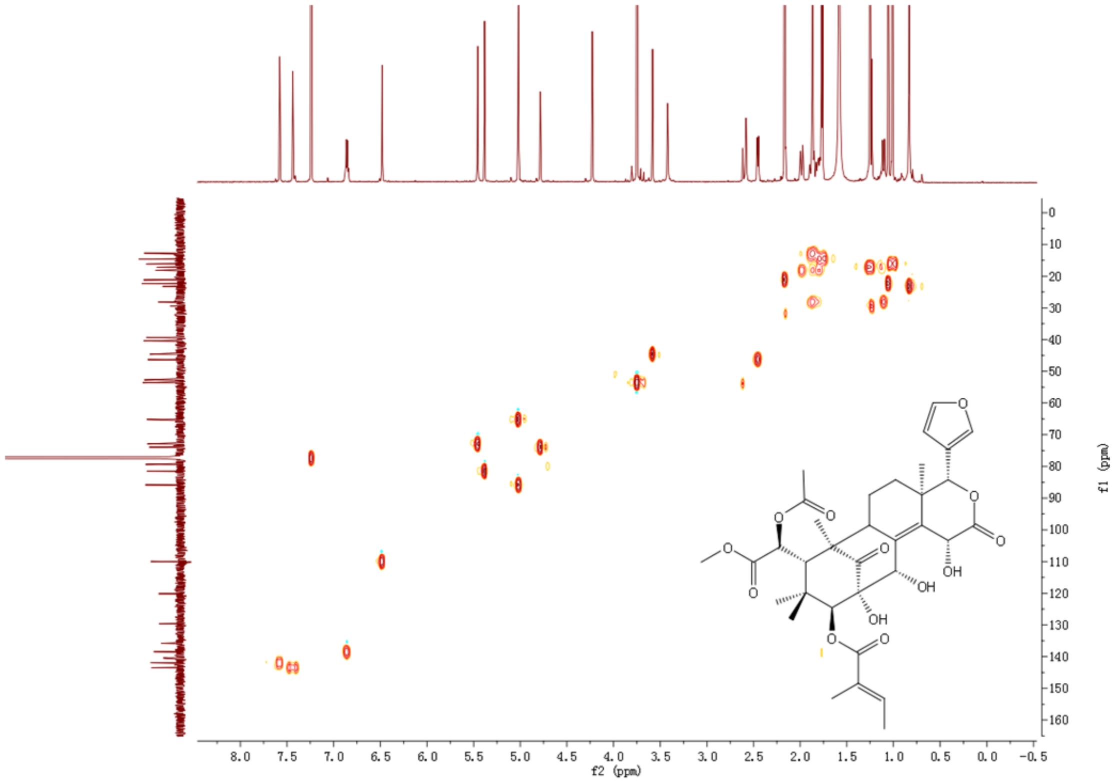

Fig. 5S HMBC NMR spectrum of compound **1** in $CDCl_3$

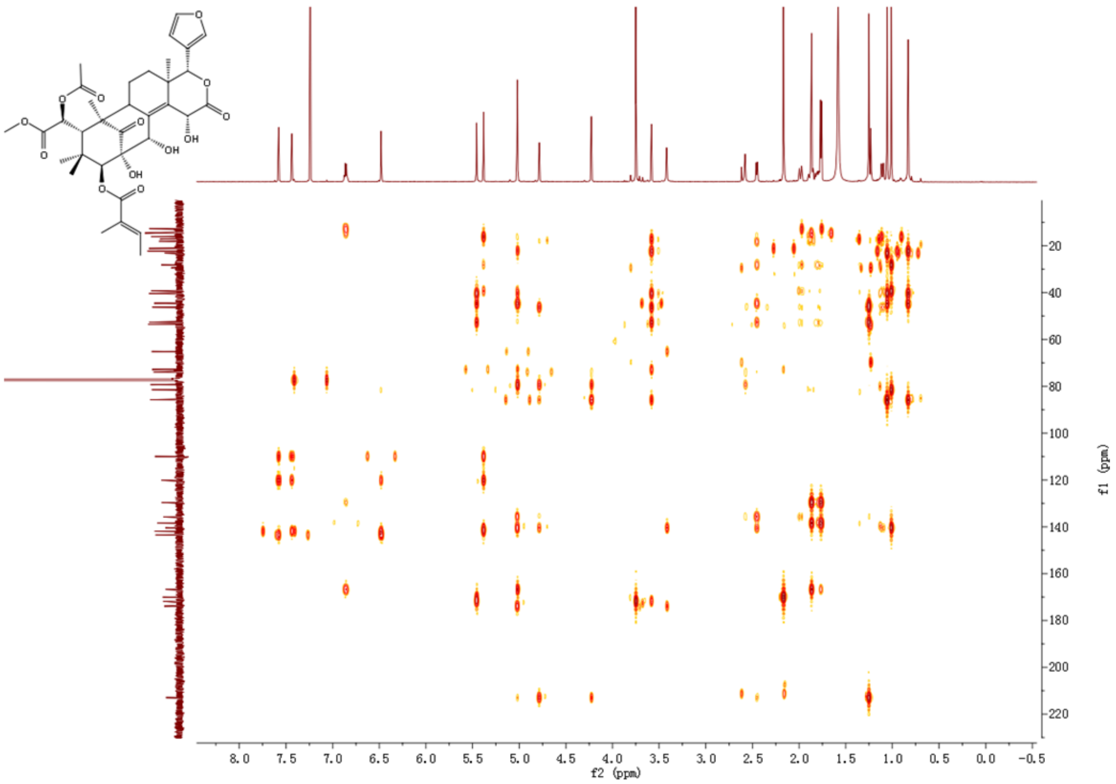

Fig. 6S ^{1}H-^{1}H COSY NMR spectrum of compound **1** in $CDCl_3$

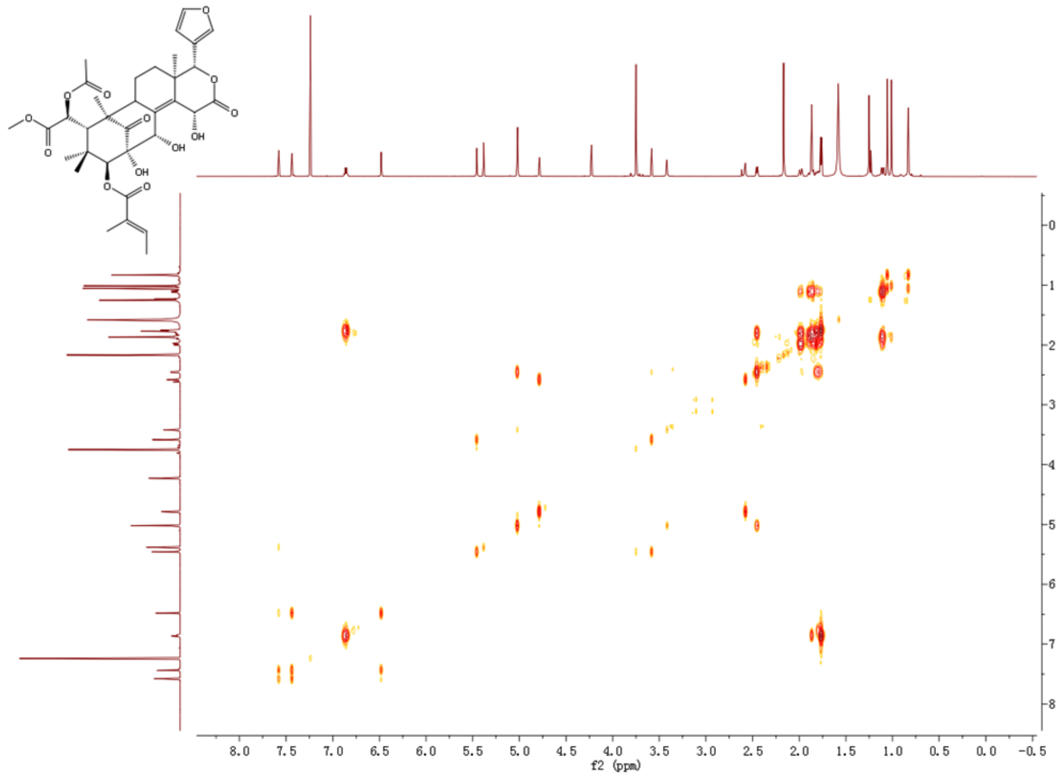

Fig. 7S ROESY NMR spectrum of compound **1** in $CDCl_3$

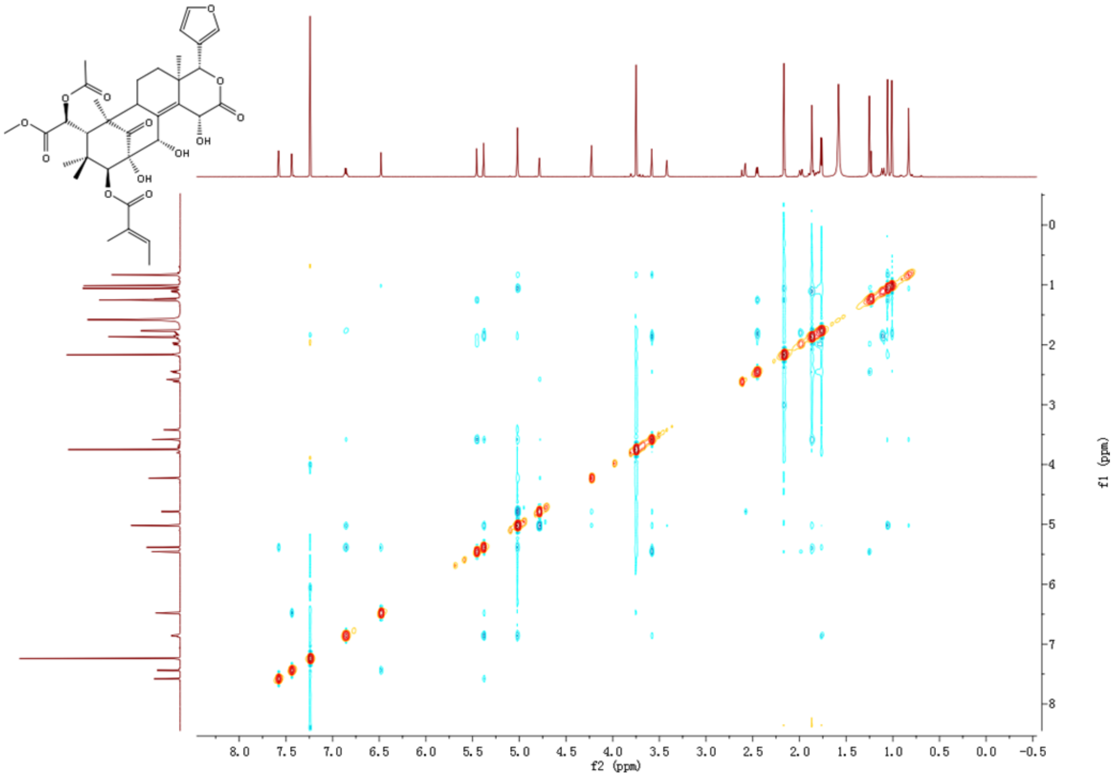

Fig. 8S IR spectrum of compound **1**

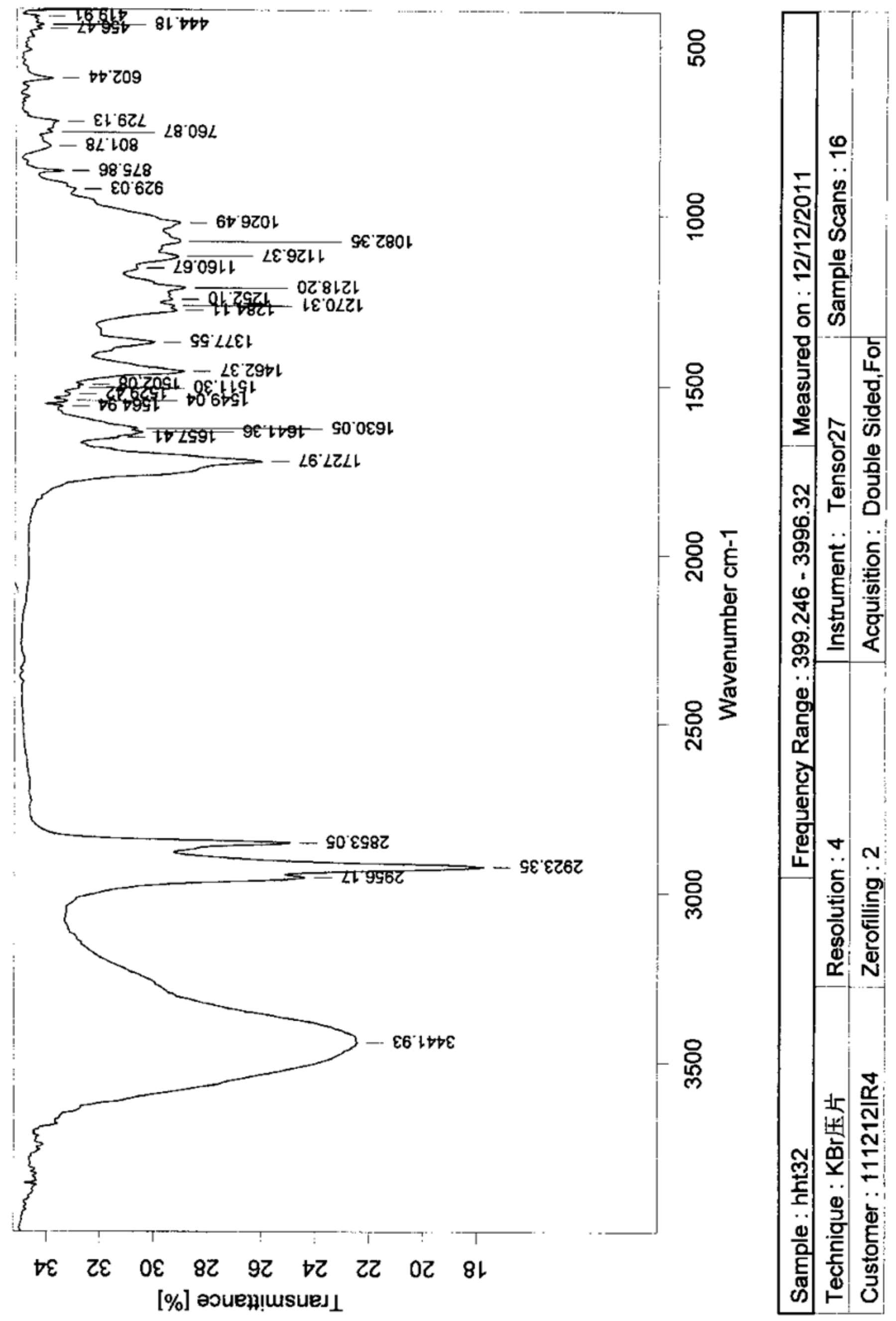

Fig. 9S UV spectrum of compound **1** in CH_3OH

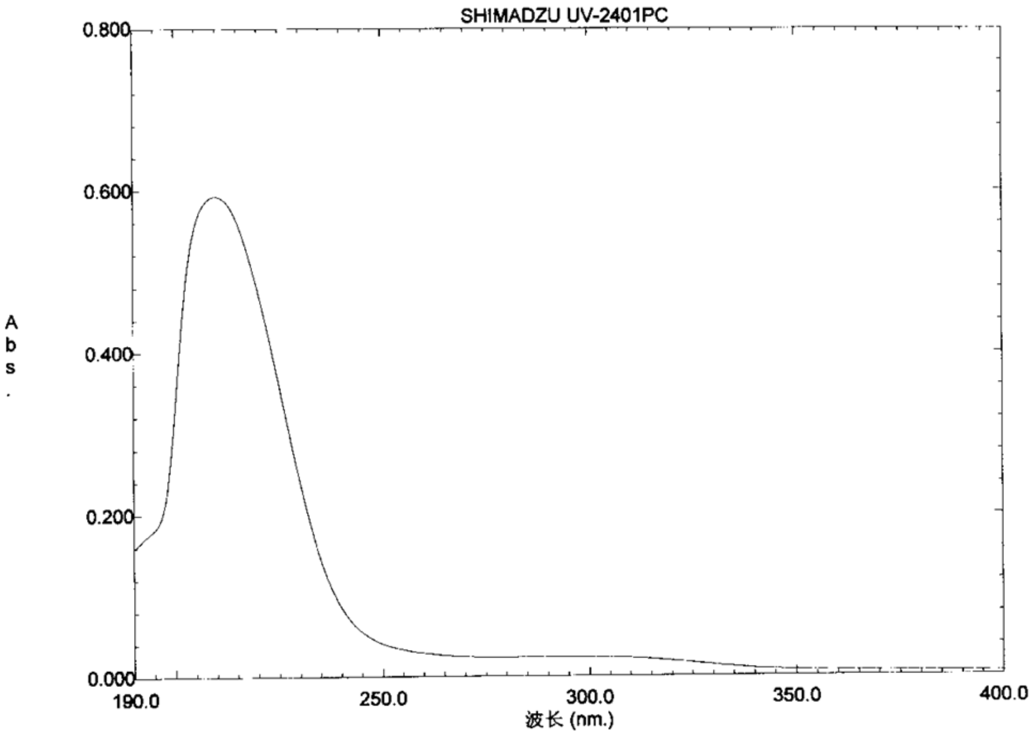

文件名: 12053102
样品名称：HHT32

创建于: 14:01 12-05-31
数据: 原始

测量模式: Abs.
扫描速度: 中速
狭缝: 5.0
采样间隔: 0.2

否.	波长 (nm.)	Abs.
1	290.20	0.0236
2	210.00	0.5921

12053102——

样品浓度：0.0202毫克/毫升
溶剂：甲醇

Fig. 10S HREIMS spectrum of compound **2**

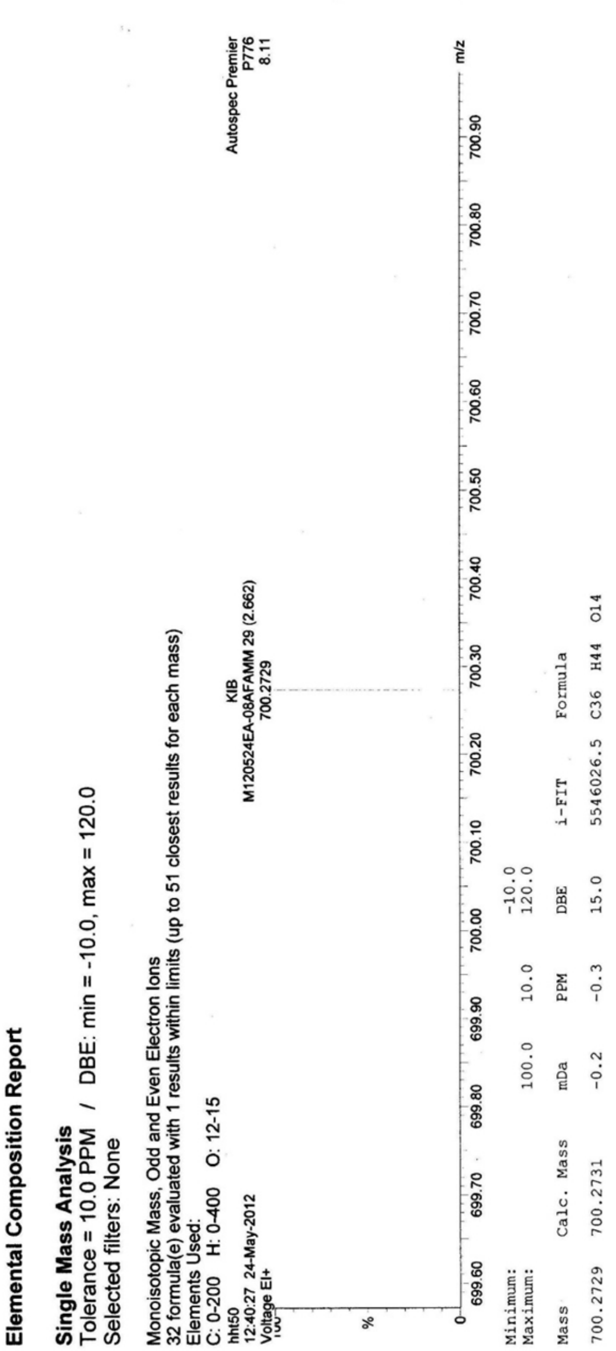

Fig. 11S ^{1}H-NMR spectrum of compound **2** in $CDCl_3$

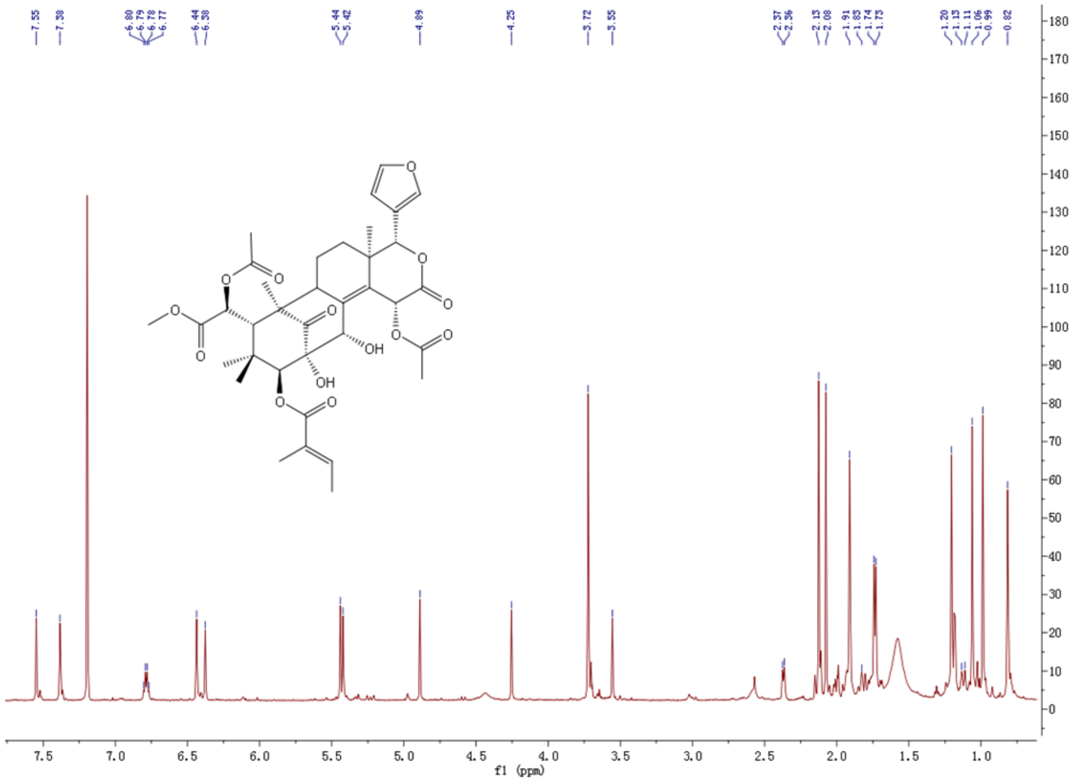

Fig. 12S ^{13}C-NMR spectrum of compound **2** in $CDCl_3$

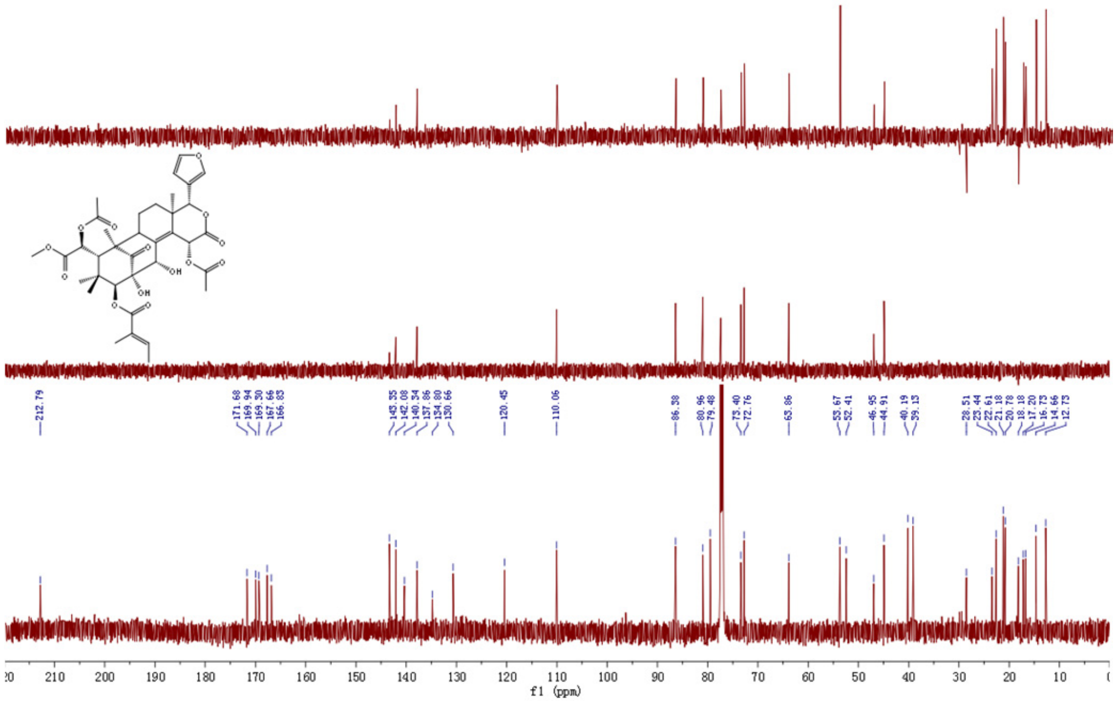

Fig. 13S HSQC NMR spectrum of compound **2** in $CDCl_3$

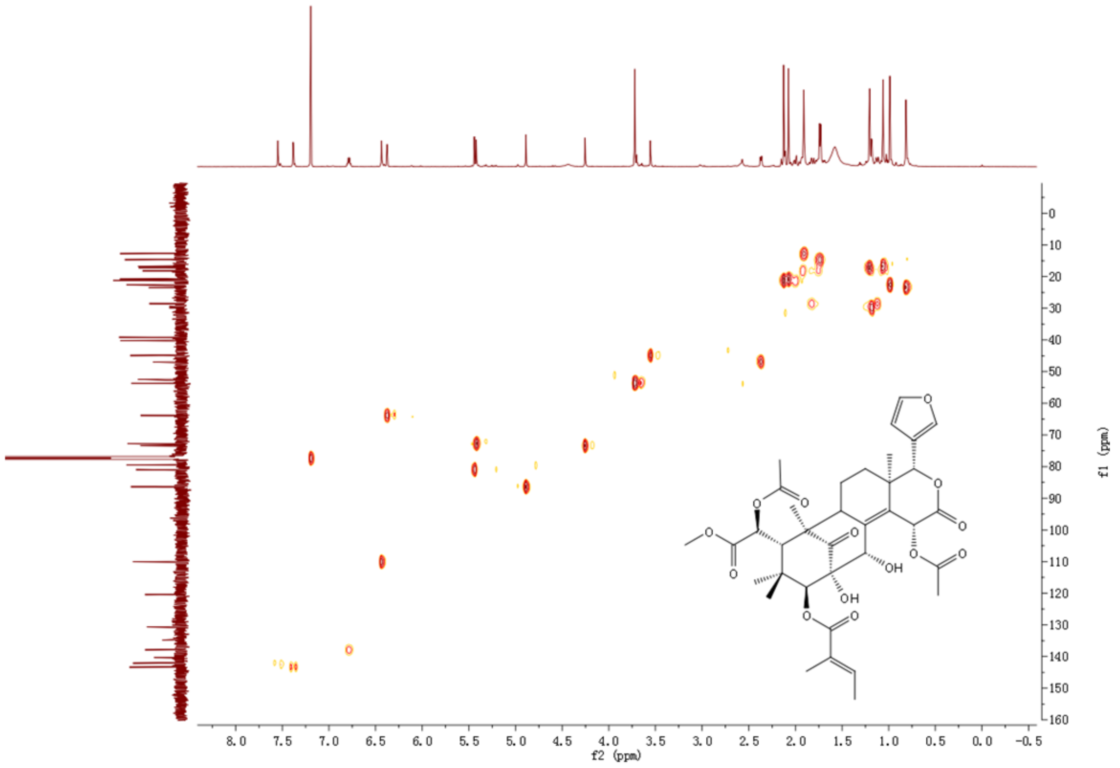

Fig. 14S HMBC NMR spectrum of compound **2** in $CDCl_3$

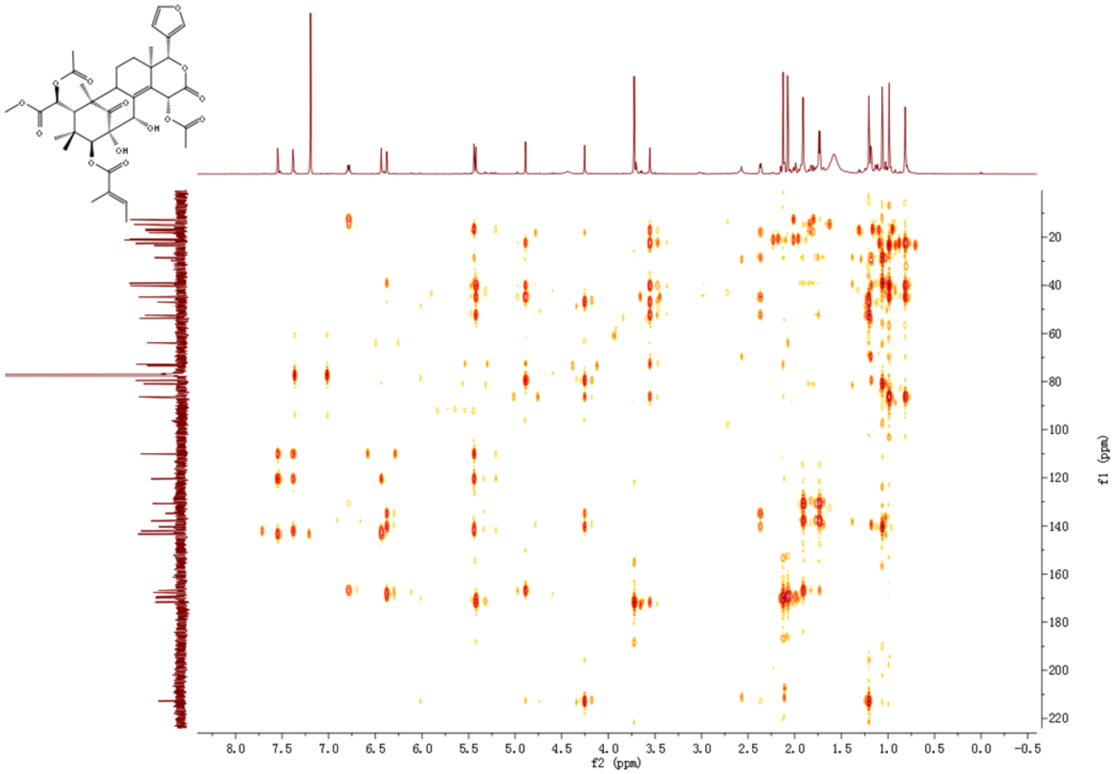

Fig. 15S ^{1}H-^{1}H COSY NMR spectrum of compound **2** in $CDCl_3$

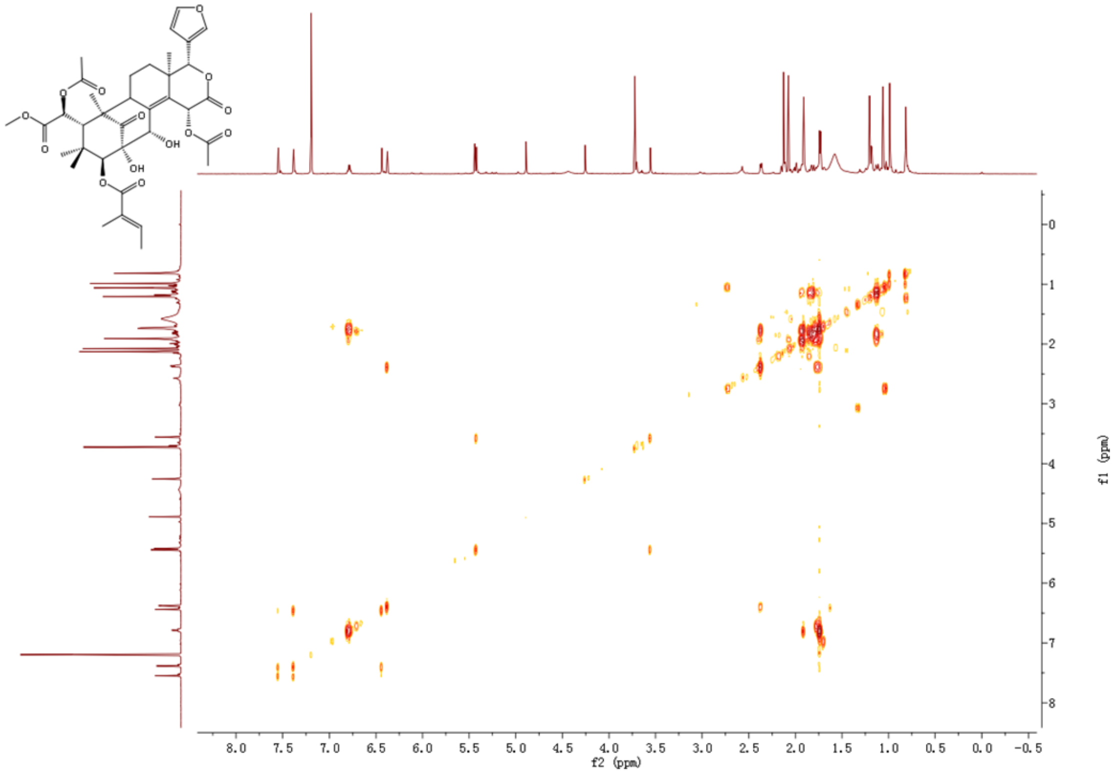

Fig. 16S ROESY NMR spectrum of compound **2** in $CDCl_3$

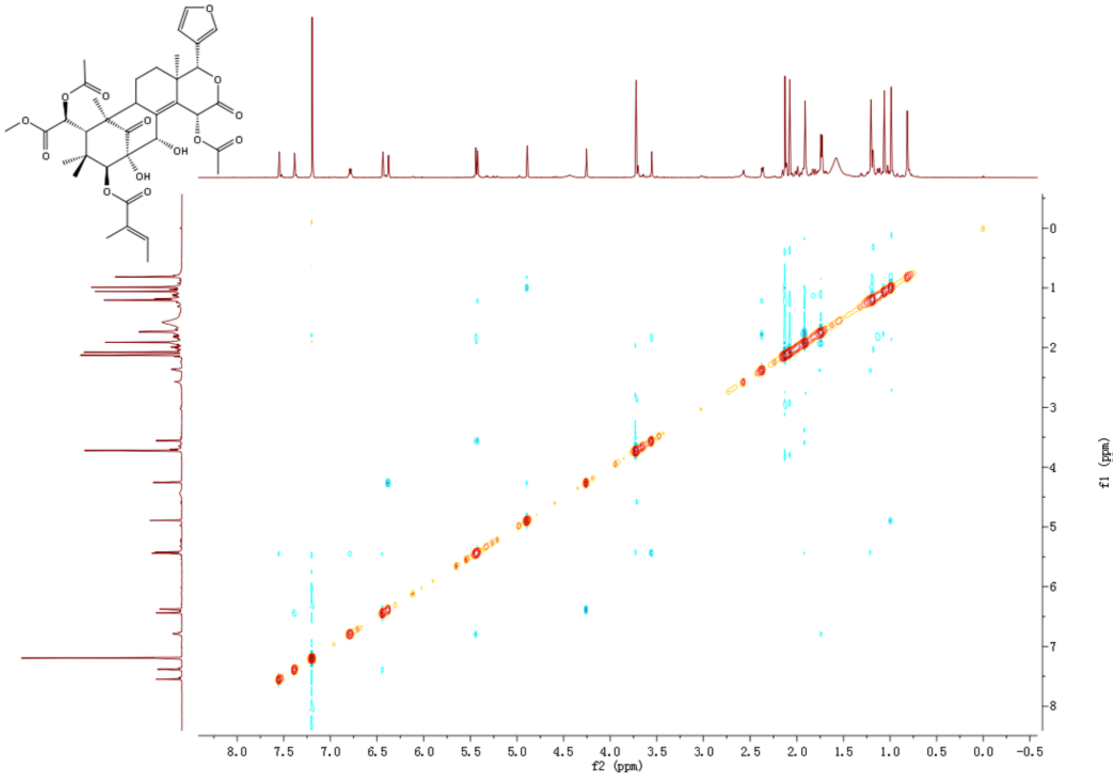

Fig. 17S IR spectrum of compound **2**

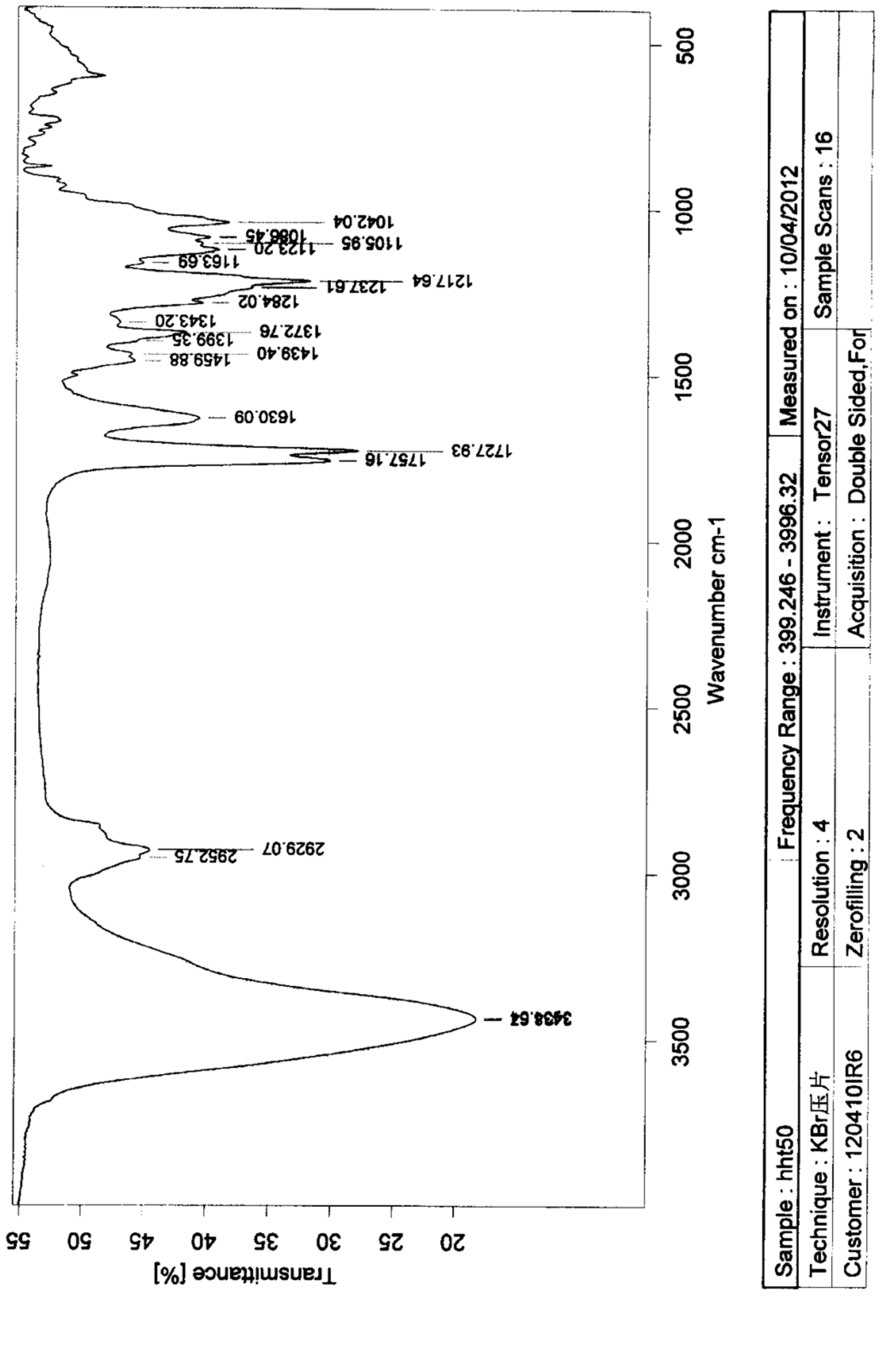

Fig. 18S UV spectrum of compound **2** in CH_3OH

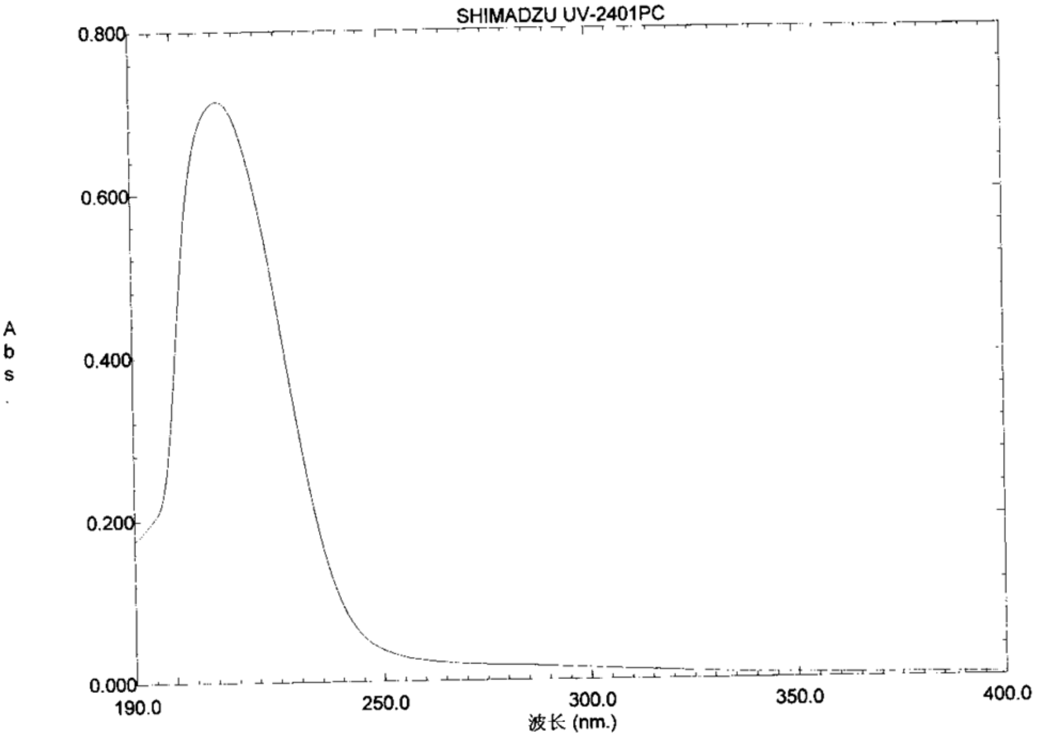

文件名: HHT50

HHT50 — —

样品浓度：0.0317毫克/毫升
溶剂：甲醇

创建于: 14:30 12-04-06
数据: 原始

测量模式: Abs.
扫描速度: 中速
狭缝: 5.0
采样间隔: 0.2

否.	波长 (nm.)	Abs.
1	211.00	0.7139

Fig. 19S HRESIMS spectrum of compound **3**

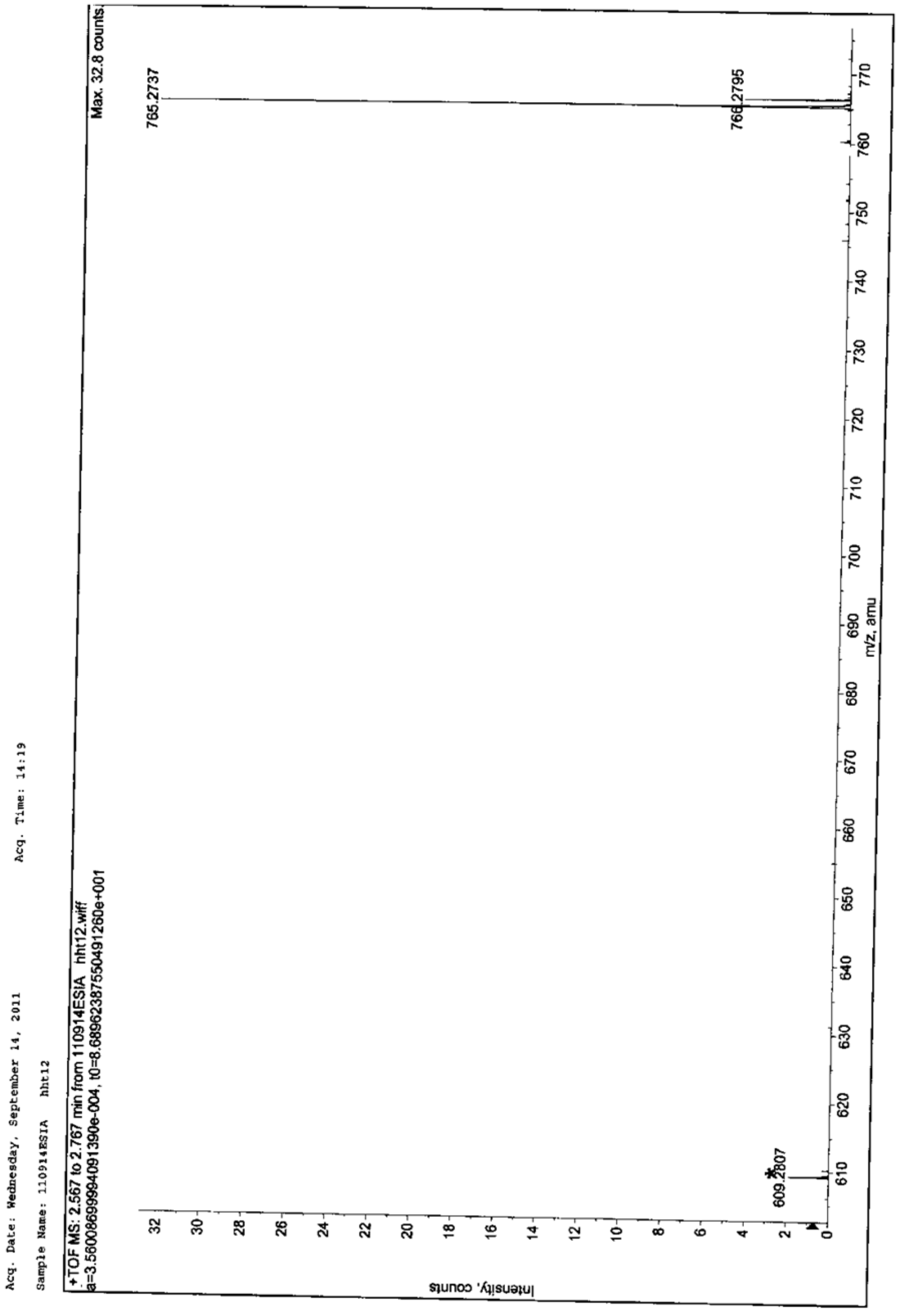

Fig. 20S ^{1}H-NMR spectrum of compound **3** in $CDCl_3$

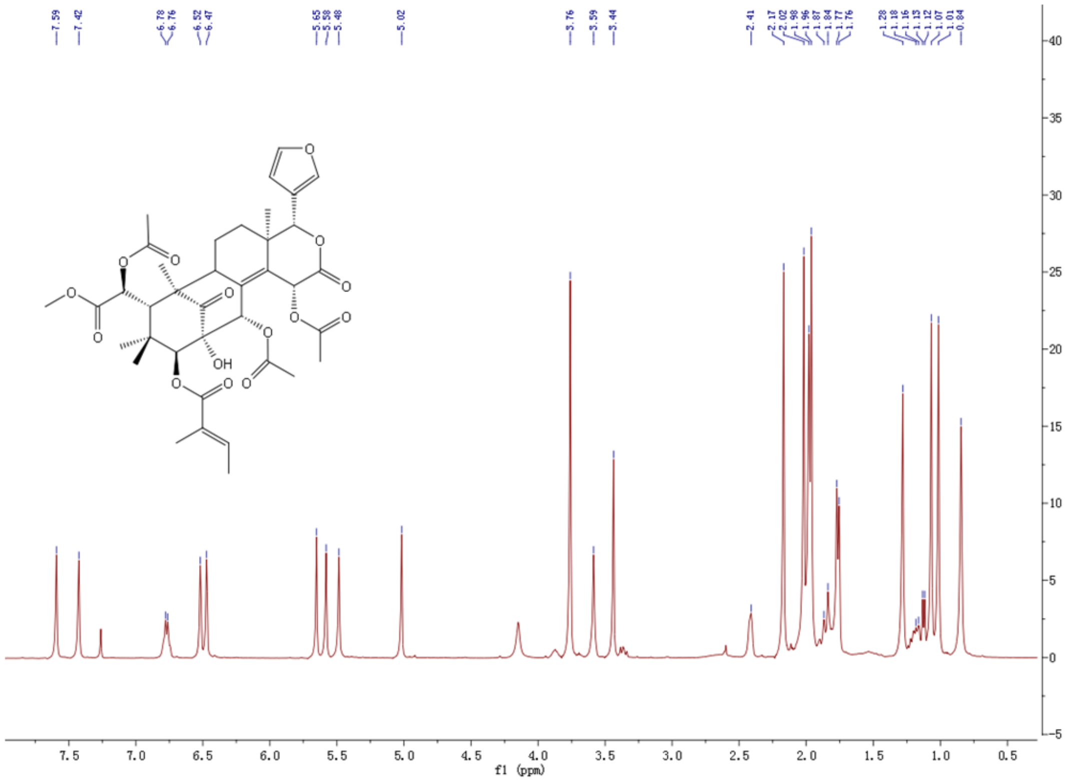

Fig. 21S ^{13}C-NMR spectrum of compound **3** in $CDCl_3$

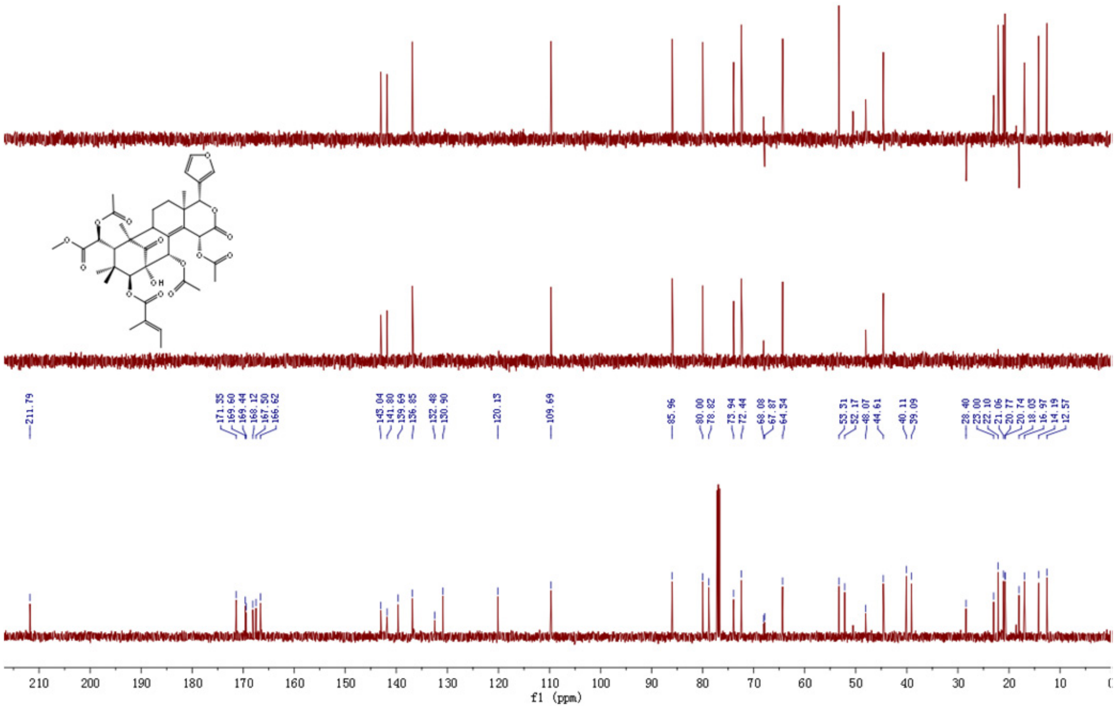

Fig. 22S HSQC NMR spectrum of compound **3** in $CDCl_3$

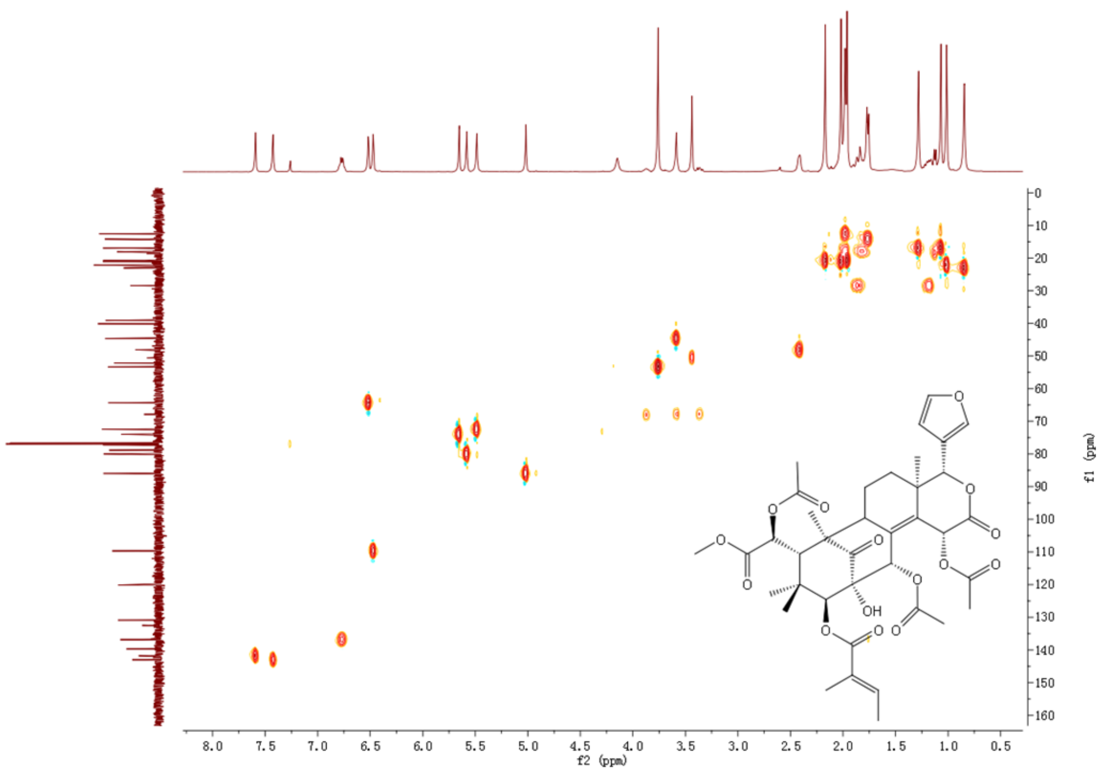

Fig. 23S HMBC NMR spectrum of compound **3** in $CDCl_3$

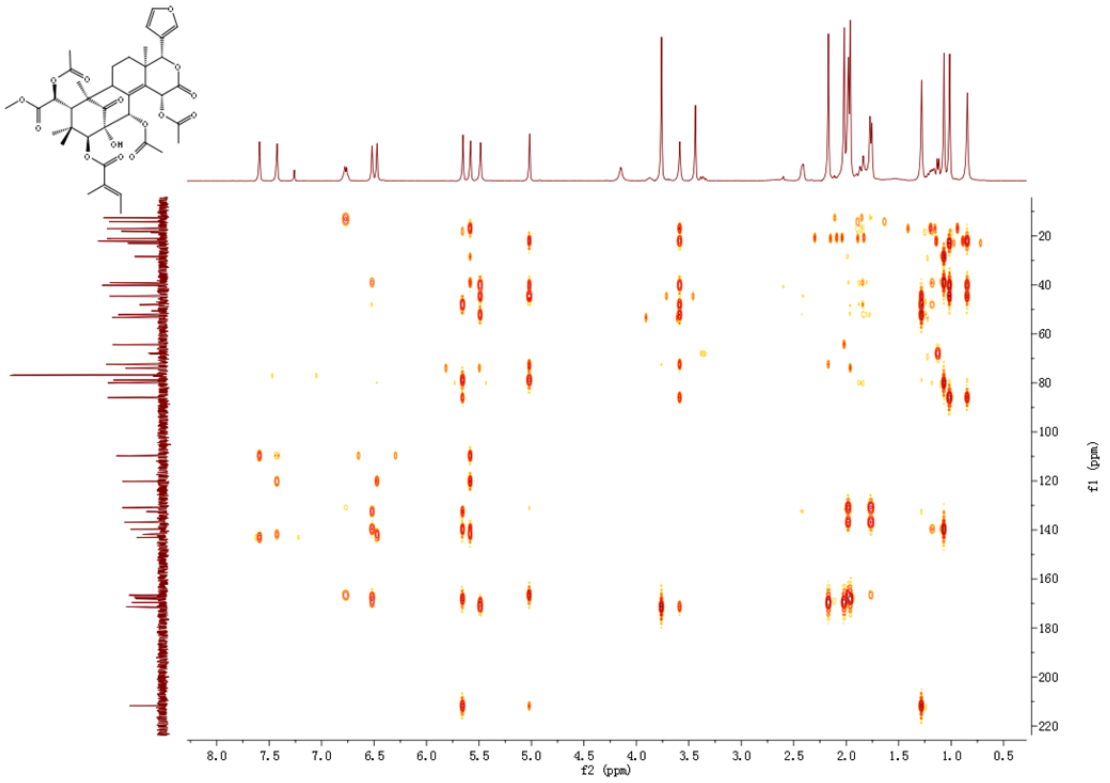

Fig. 24S ^1H-^1H COSY NMR spectrum of compound **3** in $CDCl_3$

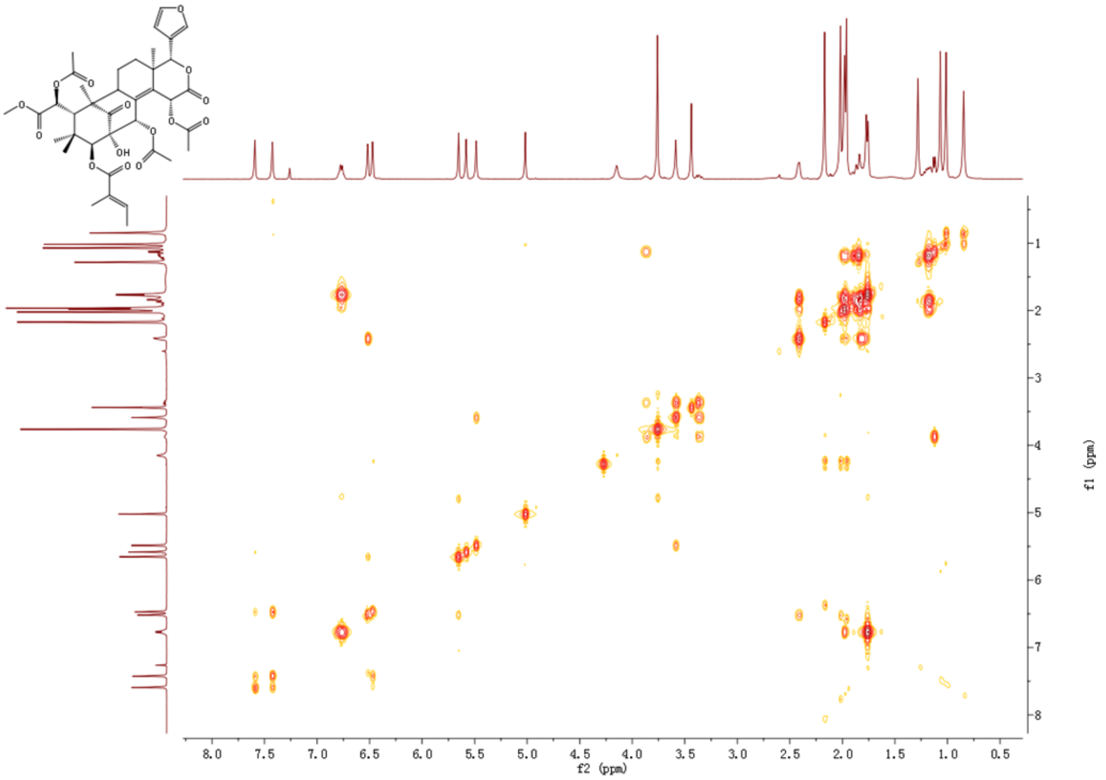

Fig. 25S ROESY NMR spectrum of compound **3** in $CDCl_3$

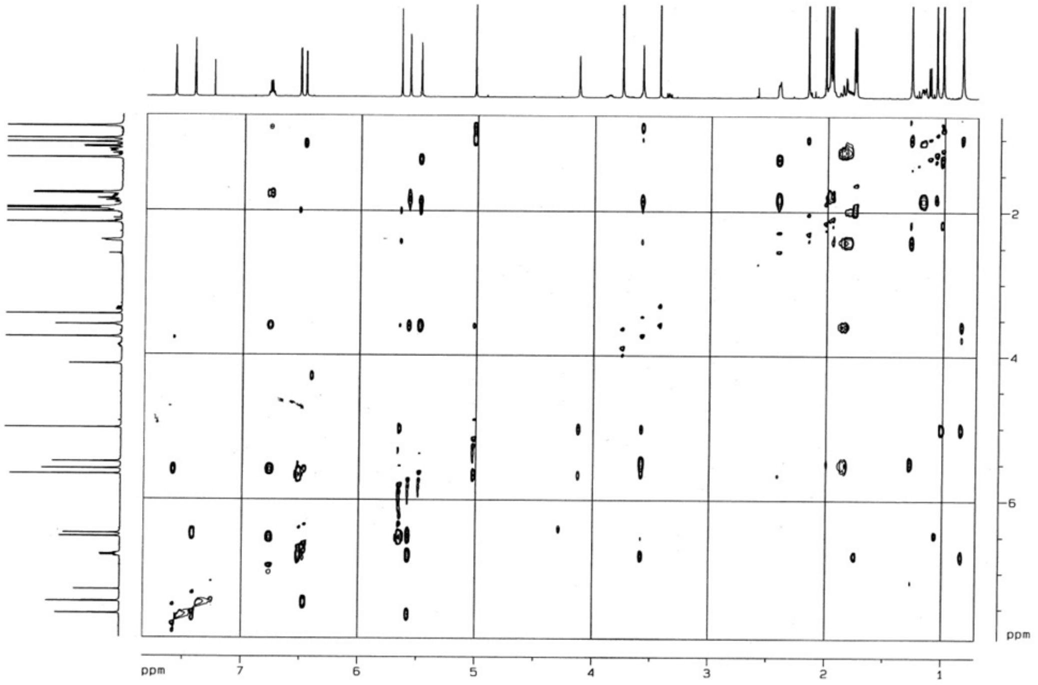

Fig. 26S IR spectrum of compound **3**

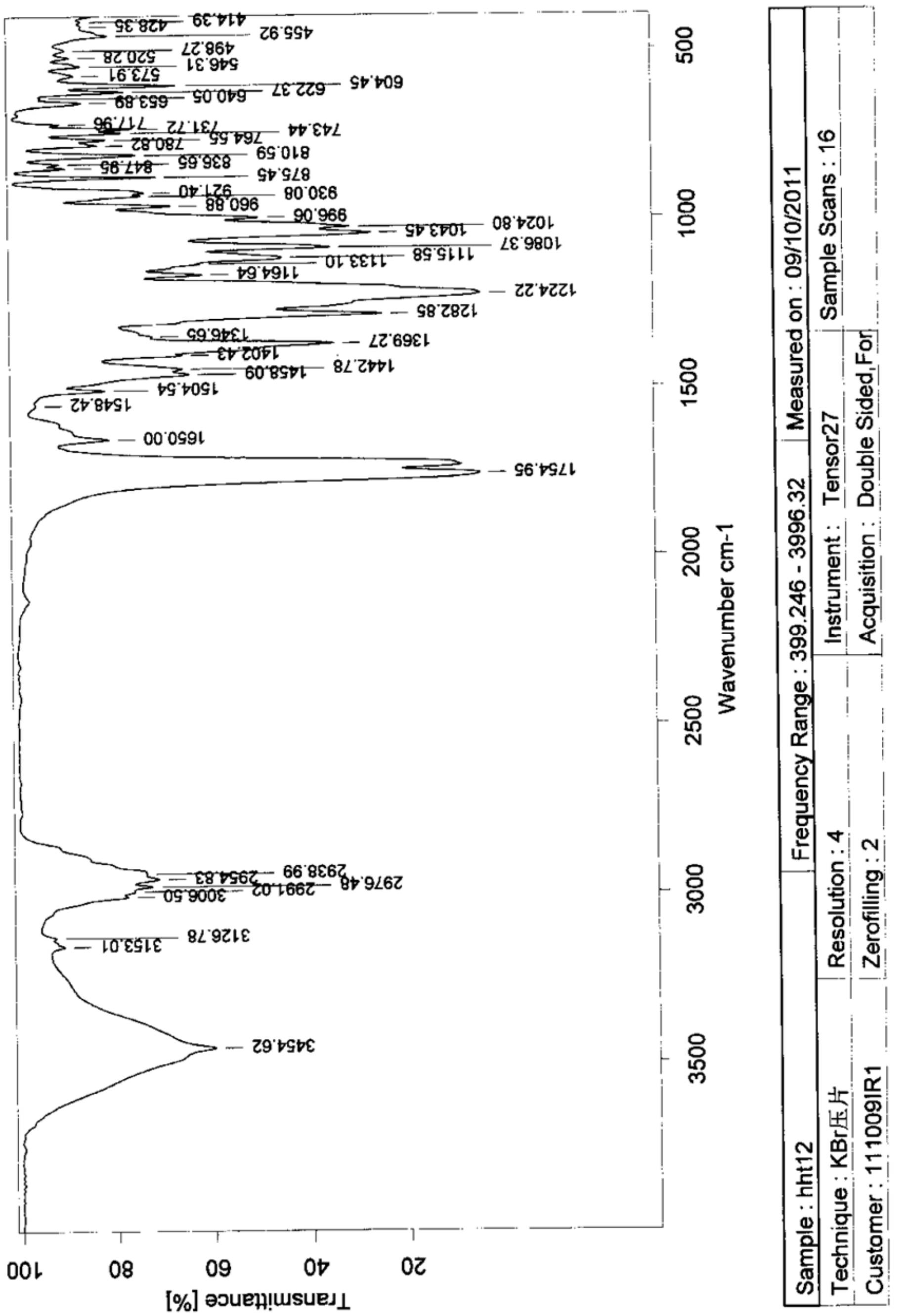

Fig. 27S UV spectrum of compound **3** in CH_3OH

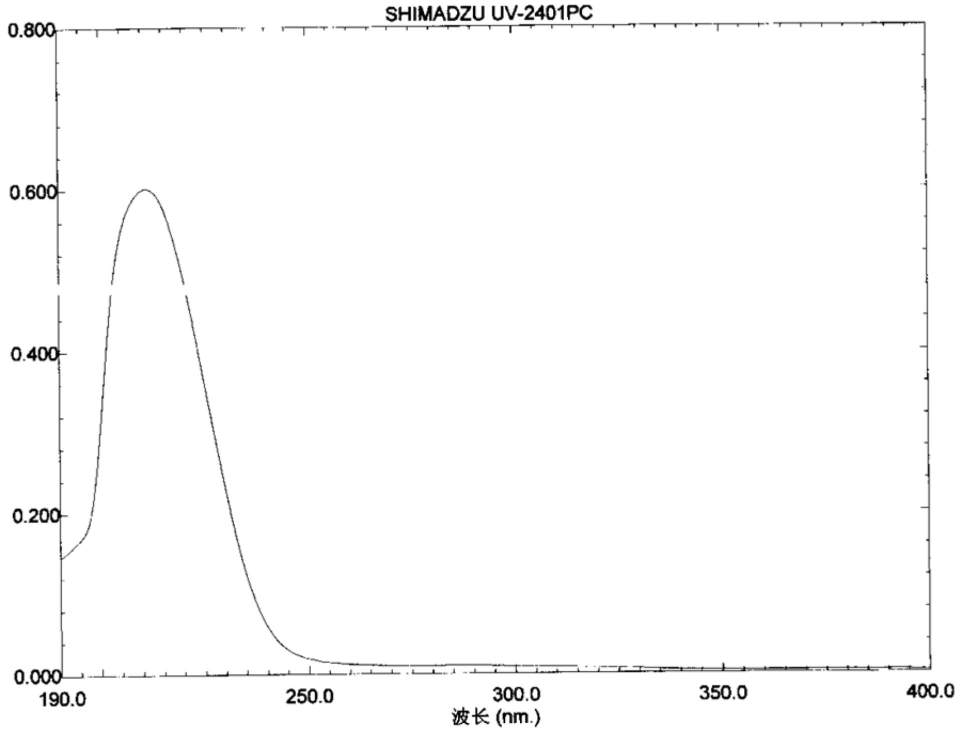

文件名:　12053100
样品名称：HHT12

创建于:　13:42　12-05-31
数据:　原始

测量模式:　Abs.
扫描速度:　中速
狭缝:　5.0
采样间隔:　0.2

否.	波长 (nm.)	Abs.
1	211.20	0.6016

12053100

样品浓度：0.0182毫克/毫升
溶剂：甲醇

Fig. 28S ESIMS spectrum of compound **4**

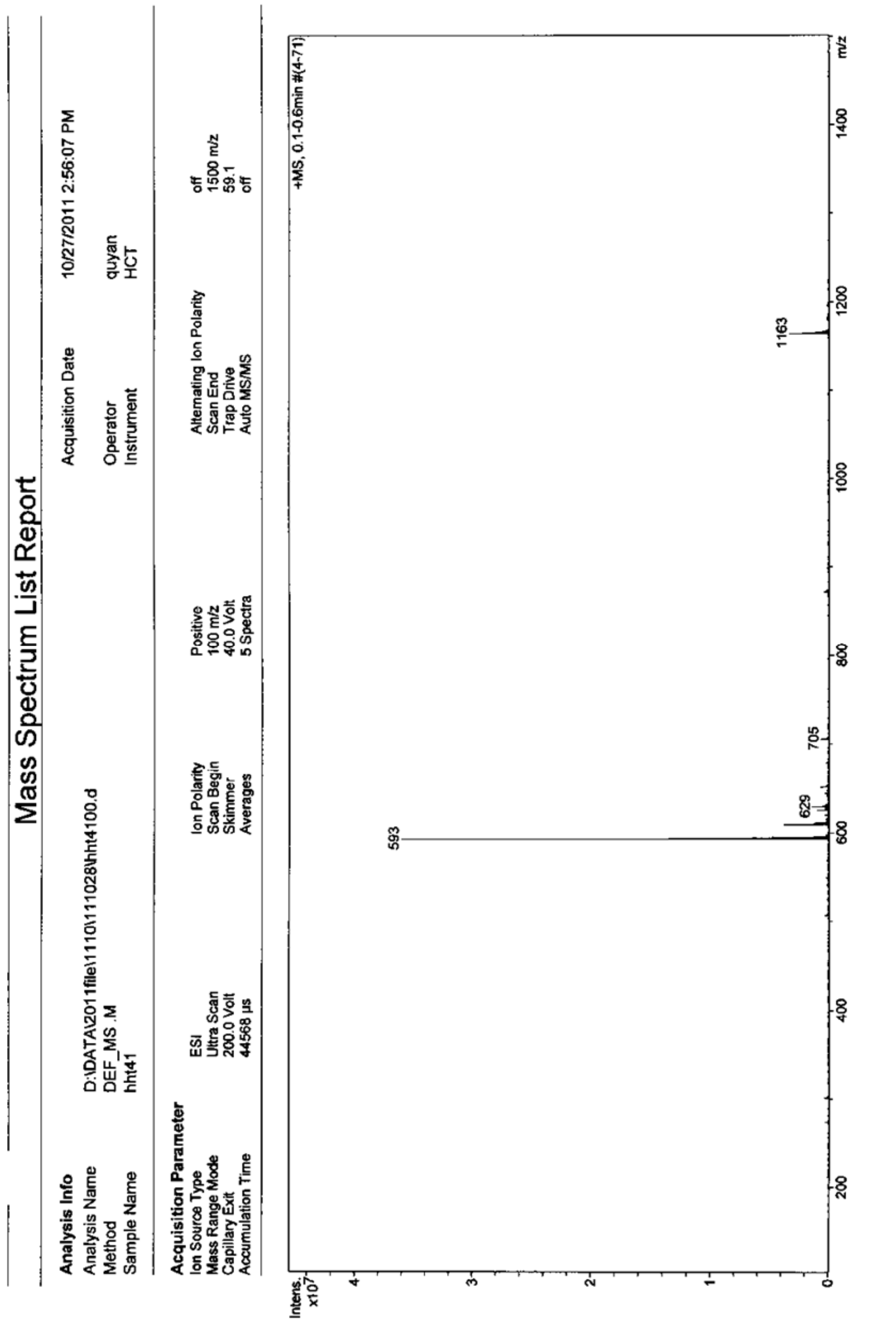

Fig. 29S ^{1}H-NMR spectrum of compound **4** in $CDCl_3$

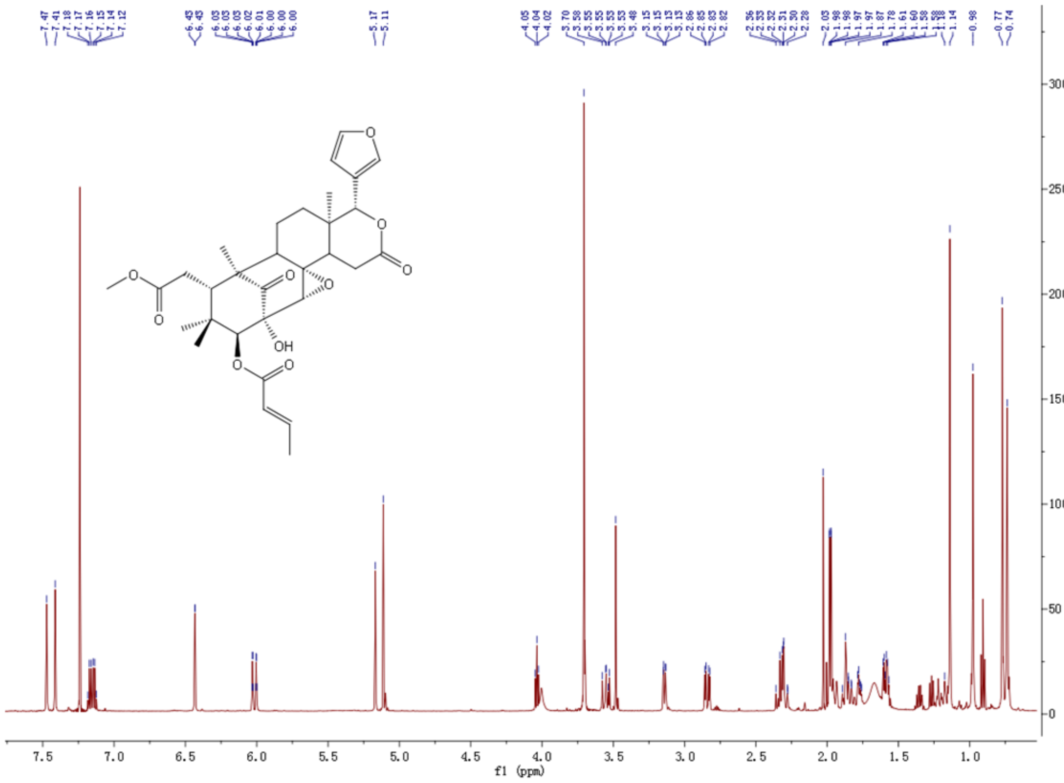

Fig. 30S ^{13}C-NMR spectrum of compound **4** in $CDCl_3$

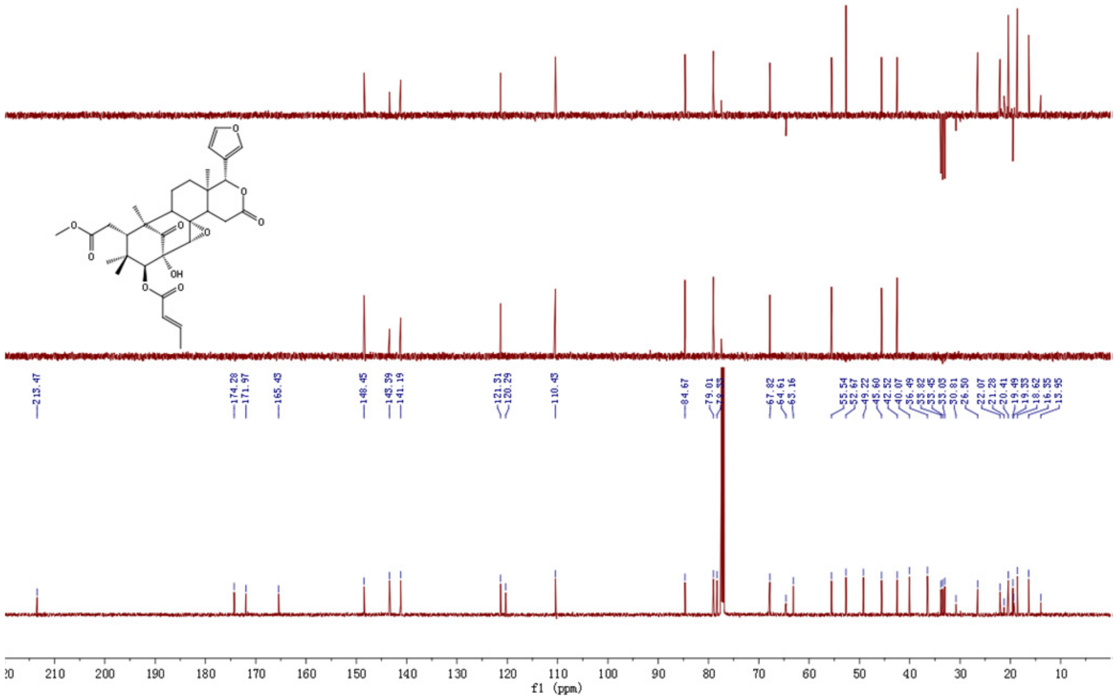

Fig. 31S HSQC NMR spectrum of compound **4** in $CDCl_3$

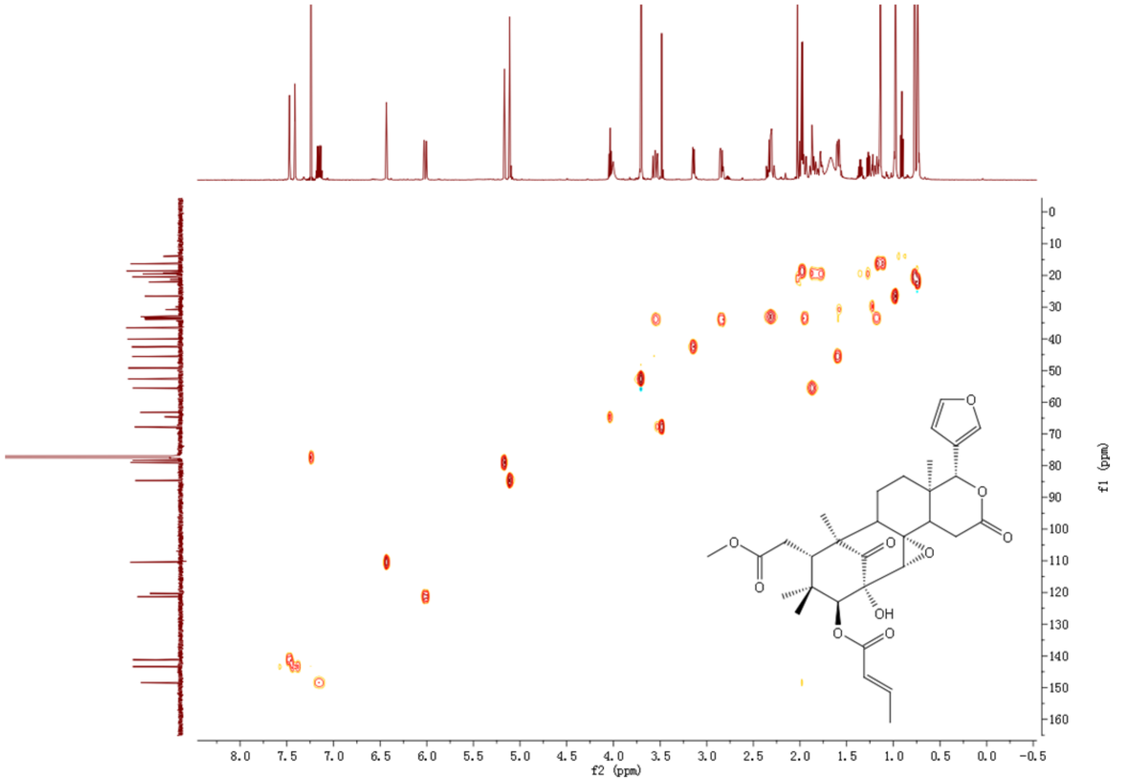

Fig. 32S HMBC NMR spectrum of compound **4** in $CDCl_3$

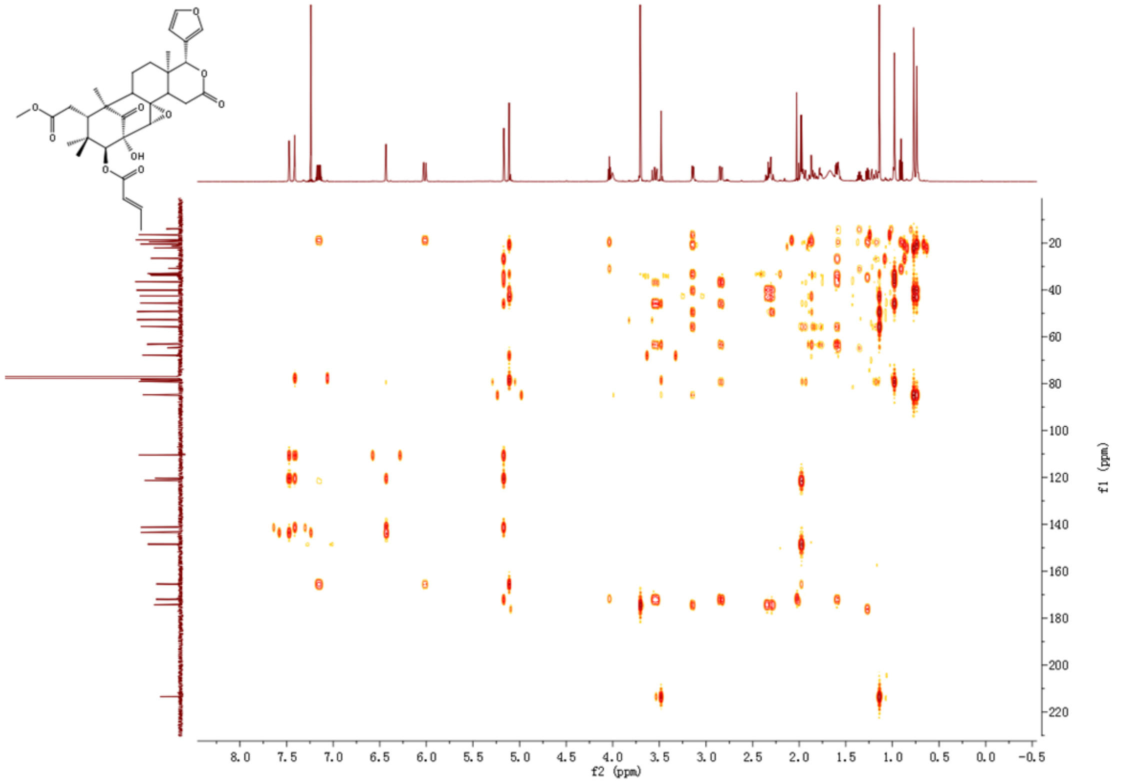

Fig. 33S ^1H-^1H COSY NMR spectrum of compound **4** in $CDCl_3$

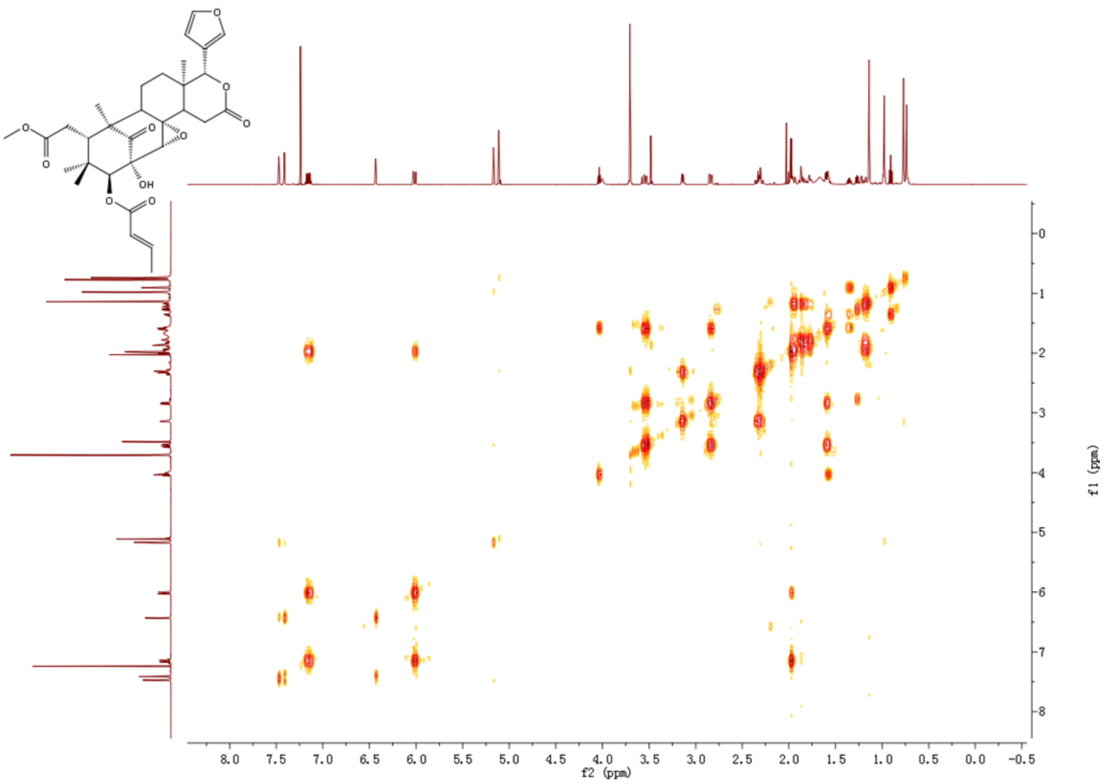

Fig. 34S ROESY NMR spectrum of compound **4** in $CDCl_3$

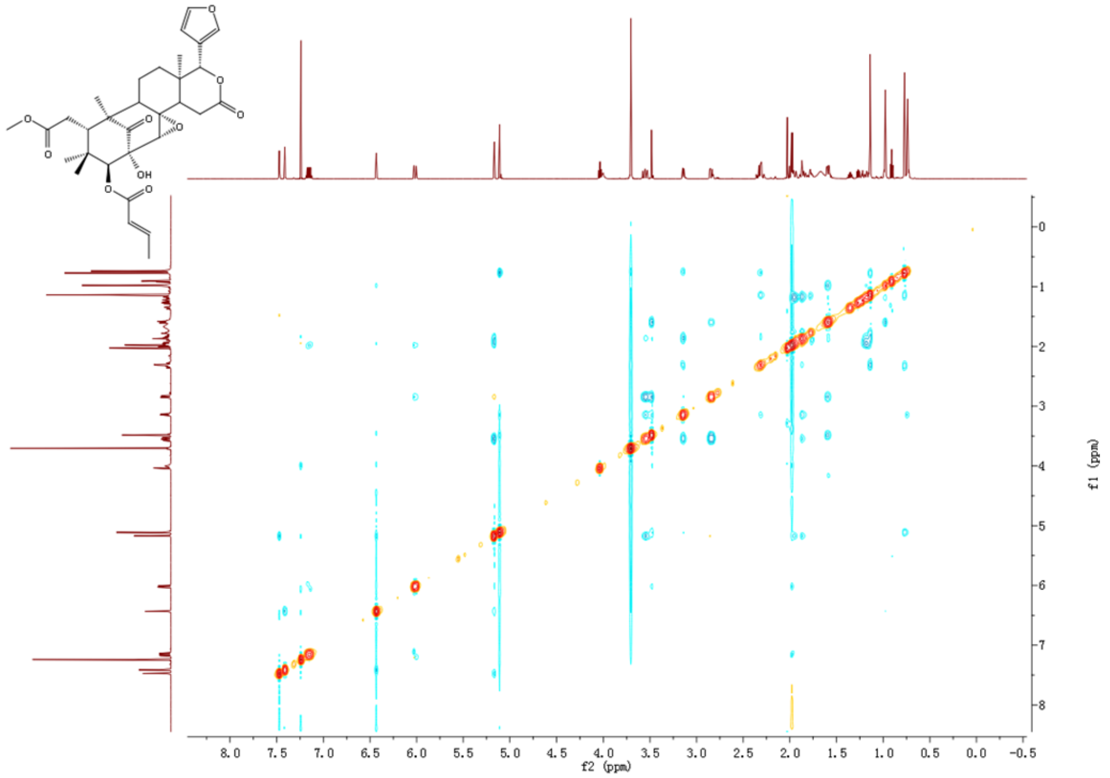

Fig. 35S IR spectrum of compound **4**

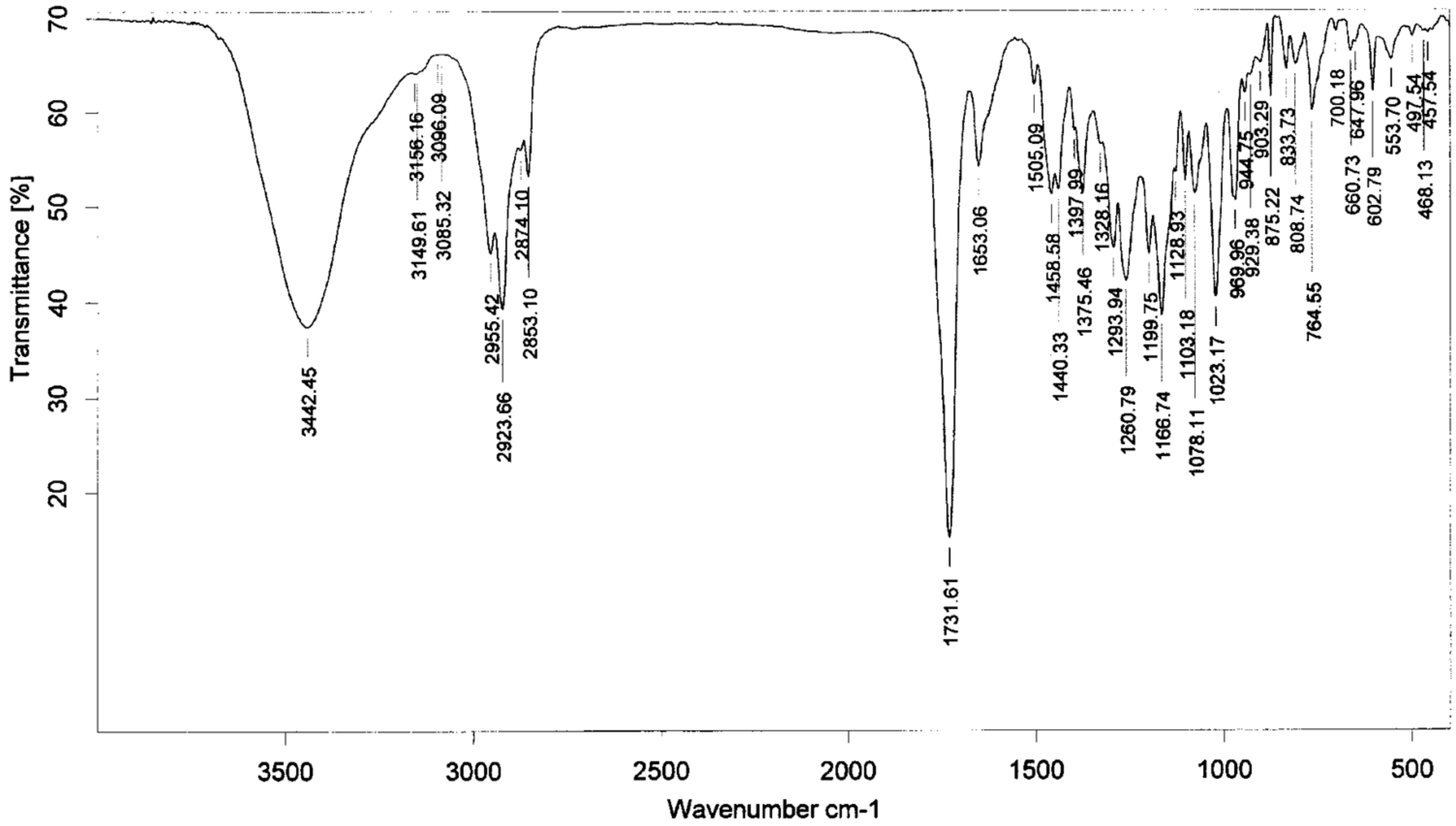

Sample : hht41	Frequency Range : 399.246 - 3996.32		Measured on : 12/12/2011
Technique : KBr压片	Resolution : 4	Instrument : Tensor27	Sample Scans : 16
Customer : 111212IR1	Zerofilling : 2	Acquisition : Double Sided,For	

Fig. 36S UV spectrum of compound **4** in CH_3OH

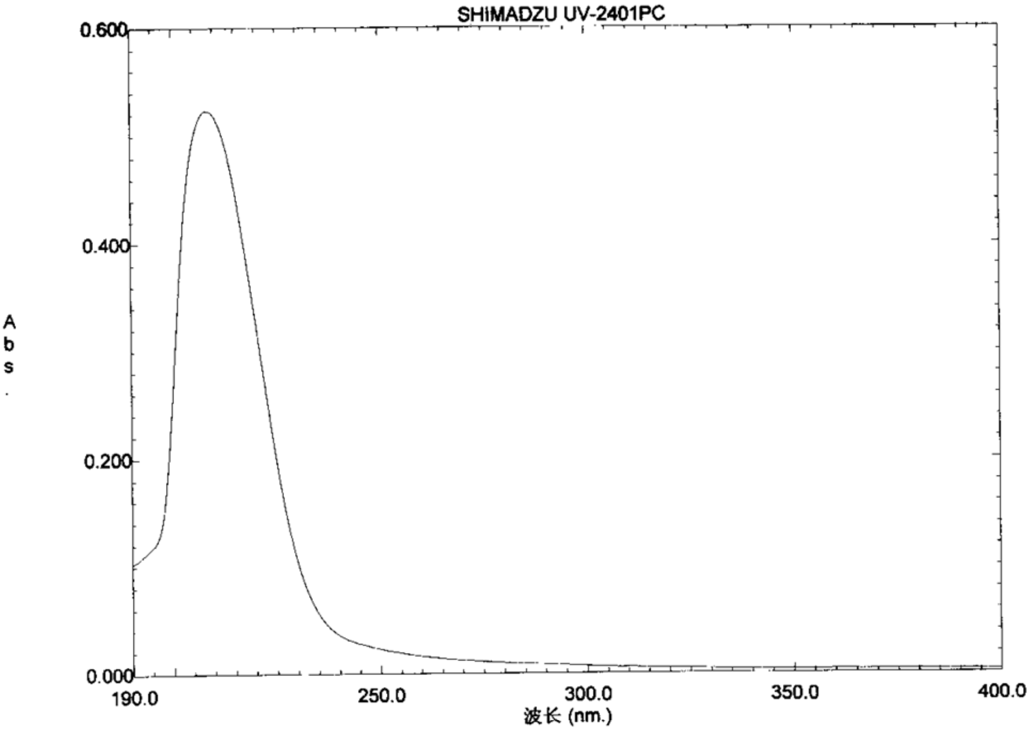

文件名: 12053101
样品名称: HHT41

创建于: 13:49 12-05-31
数据: 原始

测量模式: Abs.
扫描速度: 中速
狭缝: 5.0
采样间隔: 0.2

否.	波长 (nm.)	Abs.
1	208.40	0.5235

12053101———

样品浓度: 0.0330毫克/毫升
溶剂: 甲醇

Fig. 37S ESIMS spectrum of compound **5**

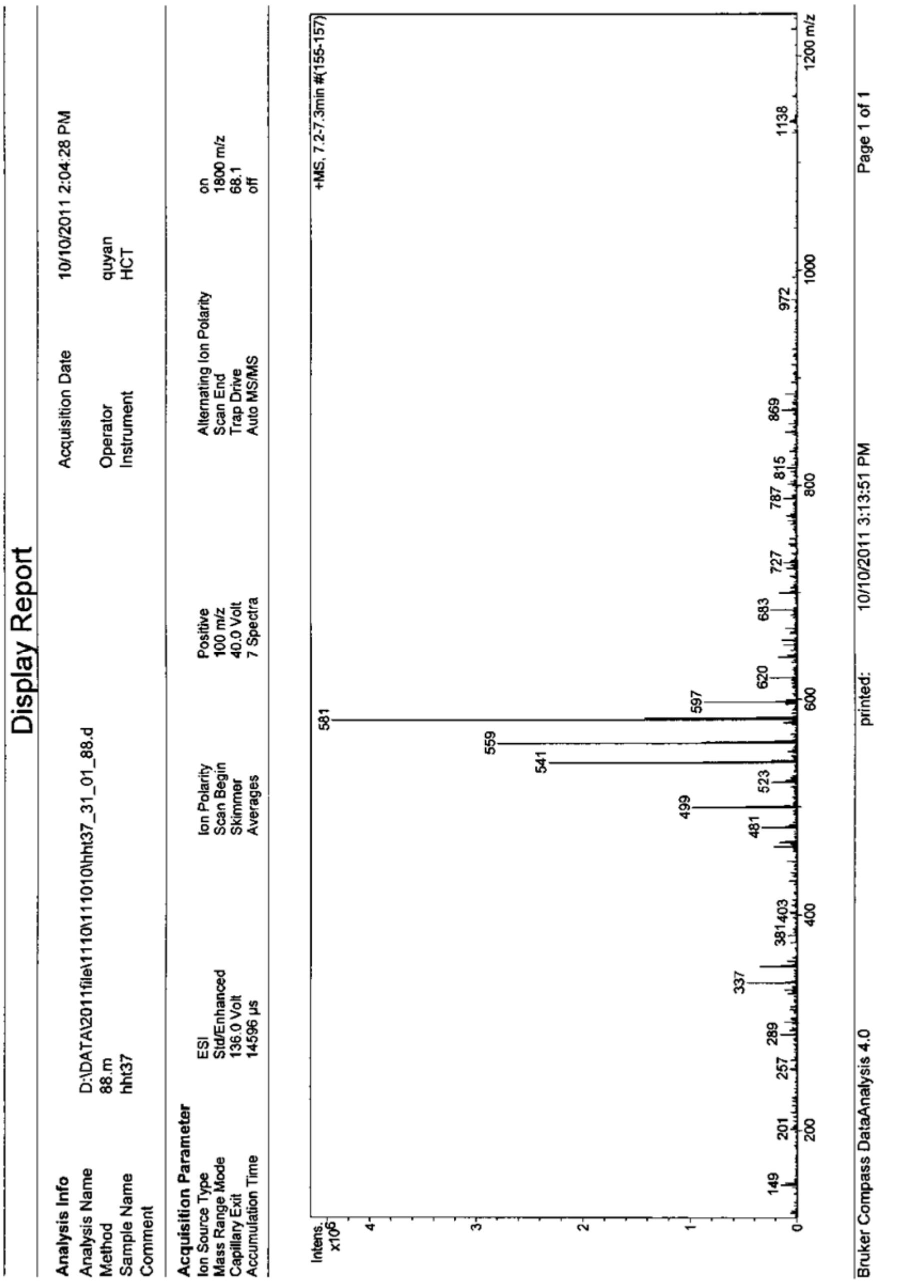

Fig. 38S ^{1}H-NMR spectrum of compound **5** in $CDCl_3$

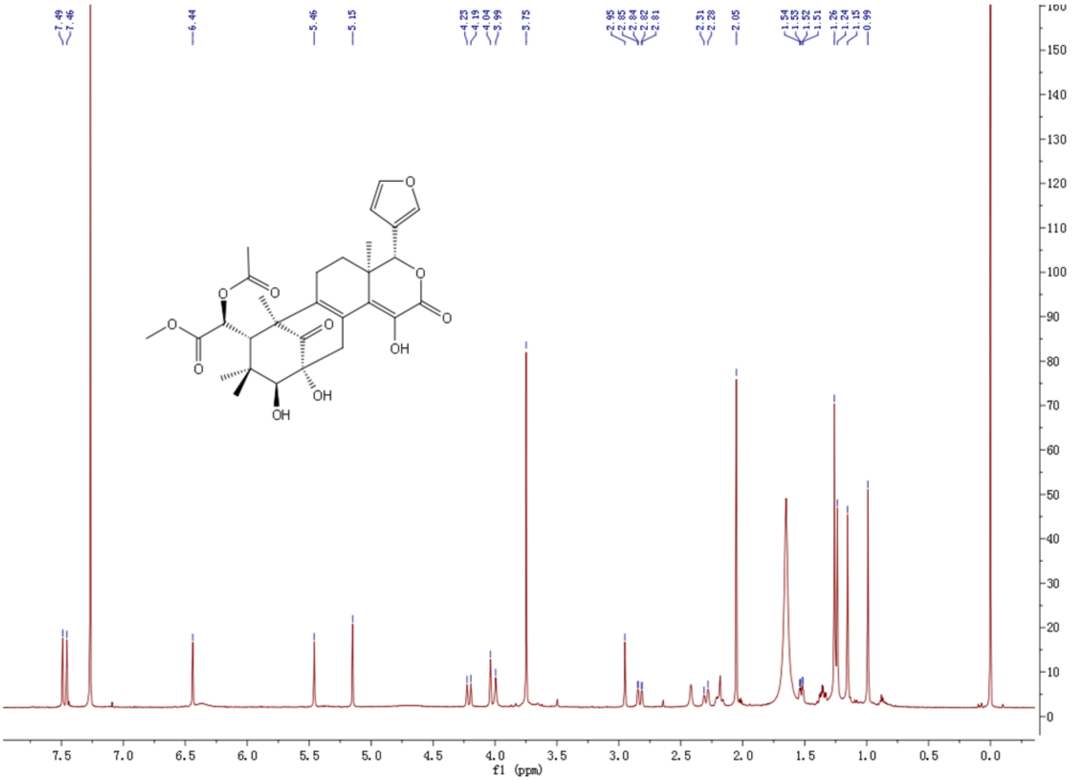

Fig. 39S ^{13}C-NMR spectrum of compound **5** in $CDCl_3$

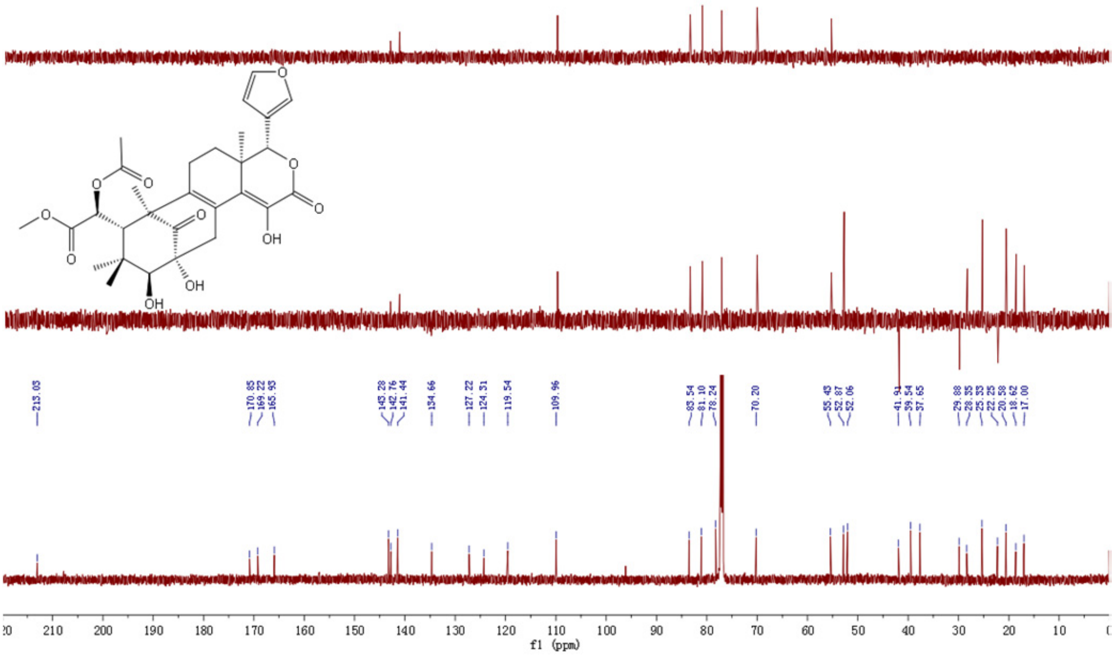

Fig. 40S HSQC NMR spectrum of compound **5** in $CDCl_3$

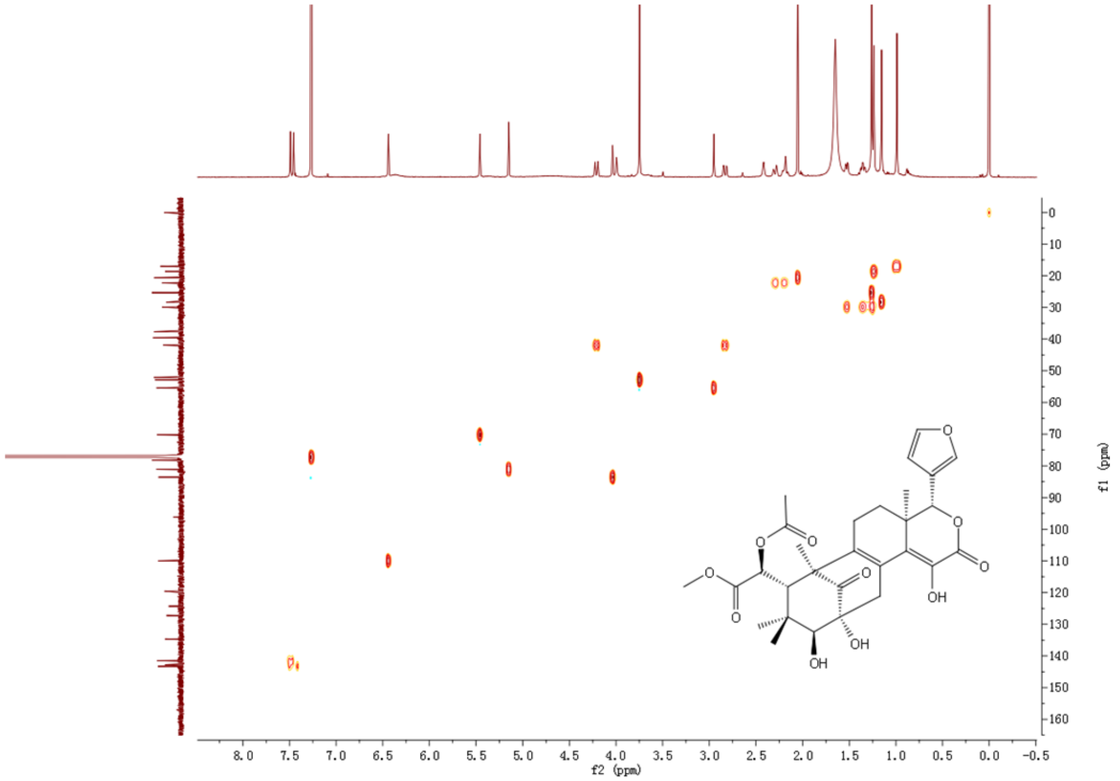

Fig. 41S HMBC NMR spectrum of compound **5** in $CDCl_3$

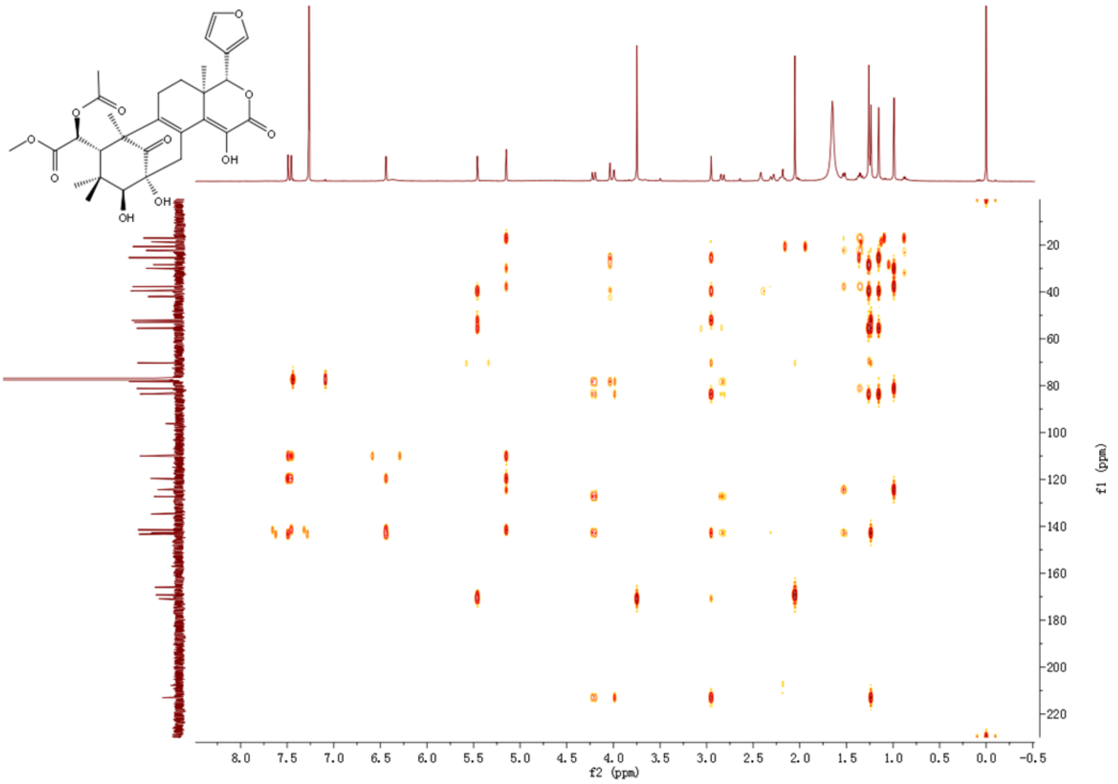

Fig. 42S ^{1}H-^{1}H COSY NMR spectrum of compound **5** in $CDCl_3$

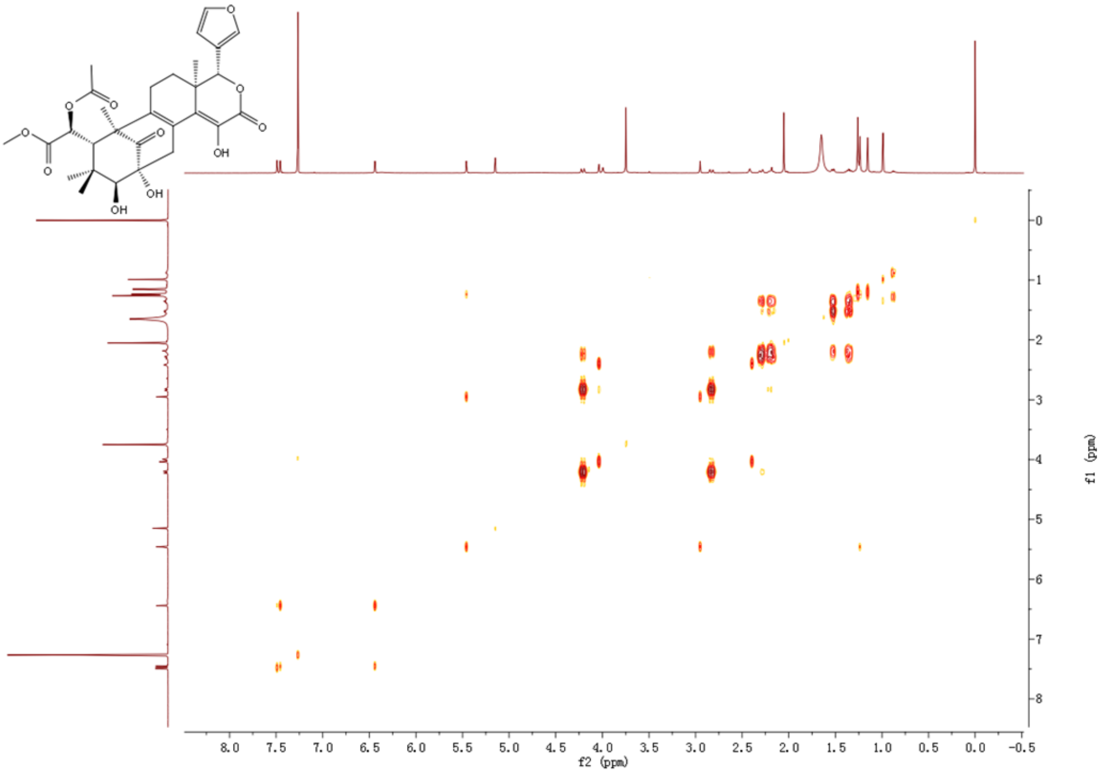

Fig. 43S ROESY NMR spectrum of compound **5** in $CDCl_3$

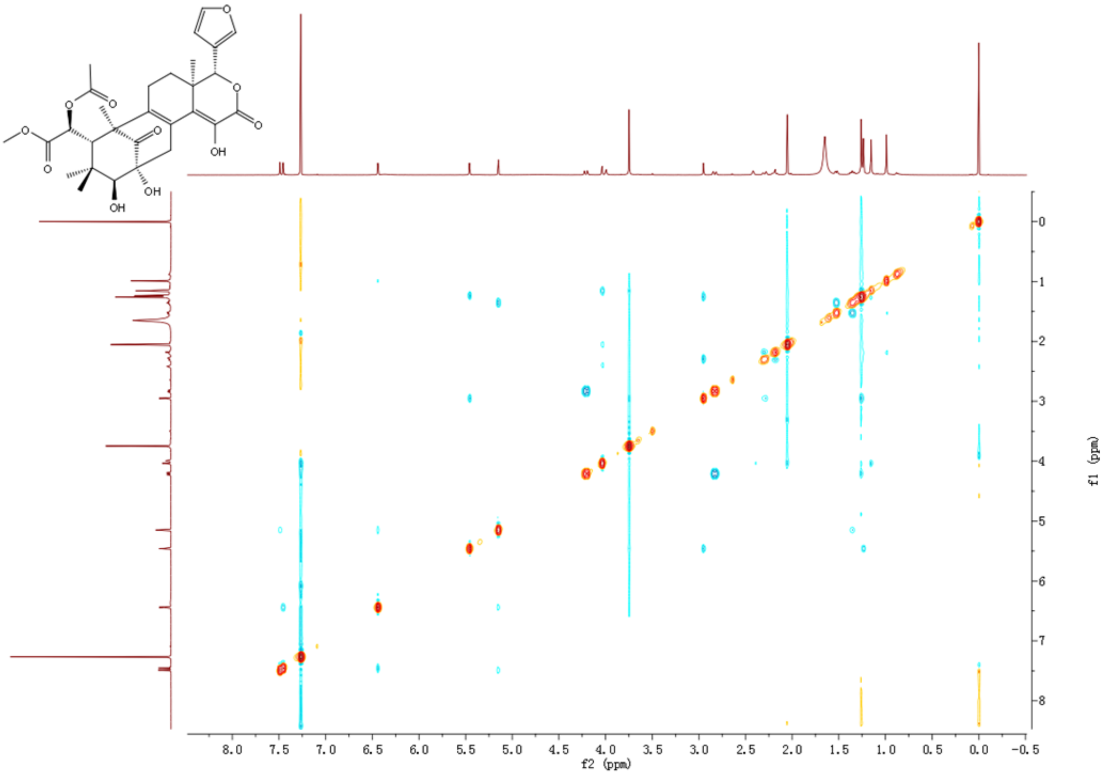

Fig. 44S IR spectrum of compound **5**

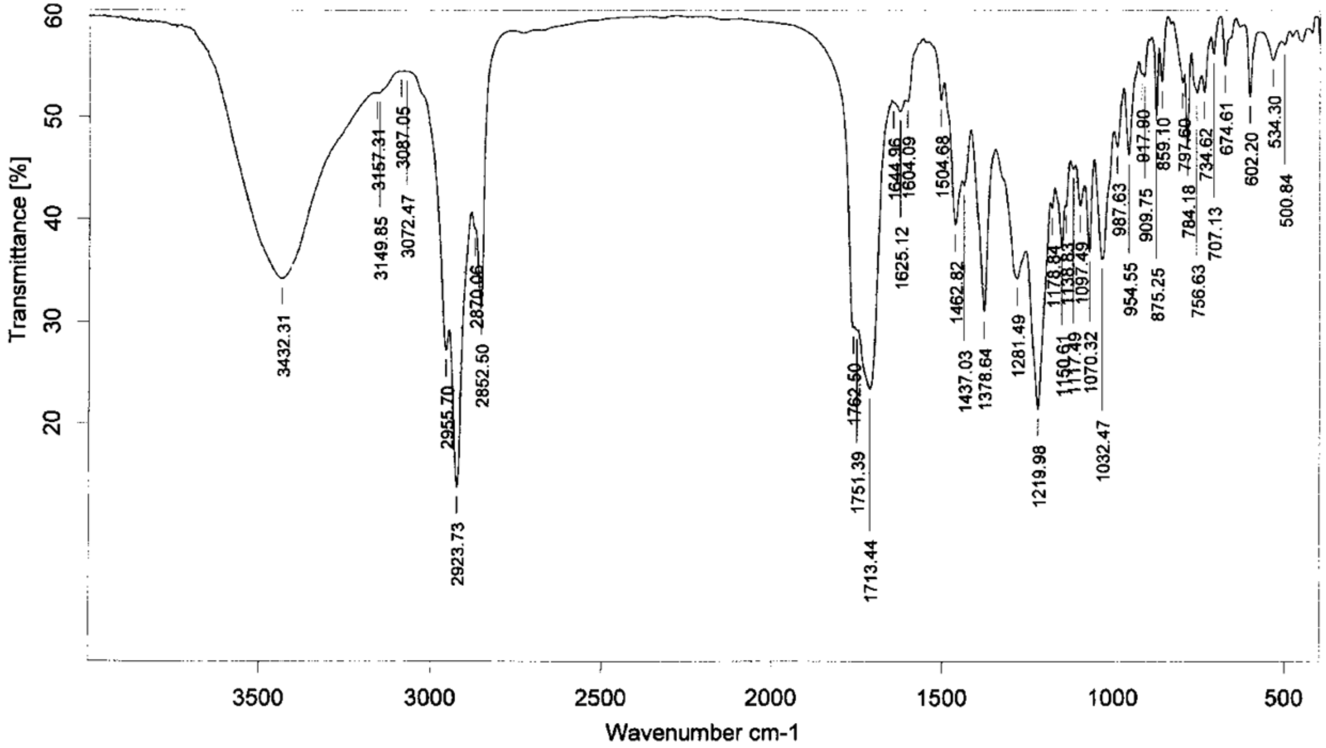

Sample : hht37	Frequency Range : 399.246 - 3996.32	Measured on : 12/12/2011	
Technique : KBr压片	Resolution : 4	Instrument : Tensor27	Sample Scans : 16
Customer : 111212IR2	Zerofilling : 2	Acquisition : Double Sided,For	
